# Supplementary material for: A comparison of DNA repair pathways to achieve a site-specific gene modification of the Bruton's tyrosine kinase gene
Source: Mol Ther Nucleic Acids. 2021 Dec 14;27:505–16. doi: 10.1016/j.omtn.2021.12.014 (PMC8728535; doi:10.1016/j.omtn.2021.12.014)
Supplement: Document S1. Figures S1–S6 [file mmc1.pdf]

OMTN, Volume *27*

Supplemental information

A comparison of DNA repair pathways to achieve a site-specific gene modification of the Bruton's tyrosine kinase gene

David H. Gray, Jasmine Santos, Alexandra Grace Keir, Isaac Villegas, Simon Maddock, Edward C. Trope, Joseph D. Long, and Caroline Y. Kuo

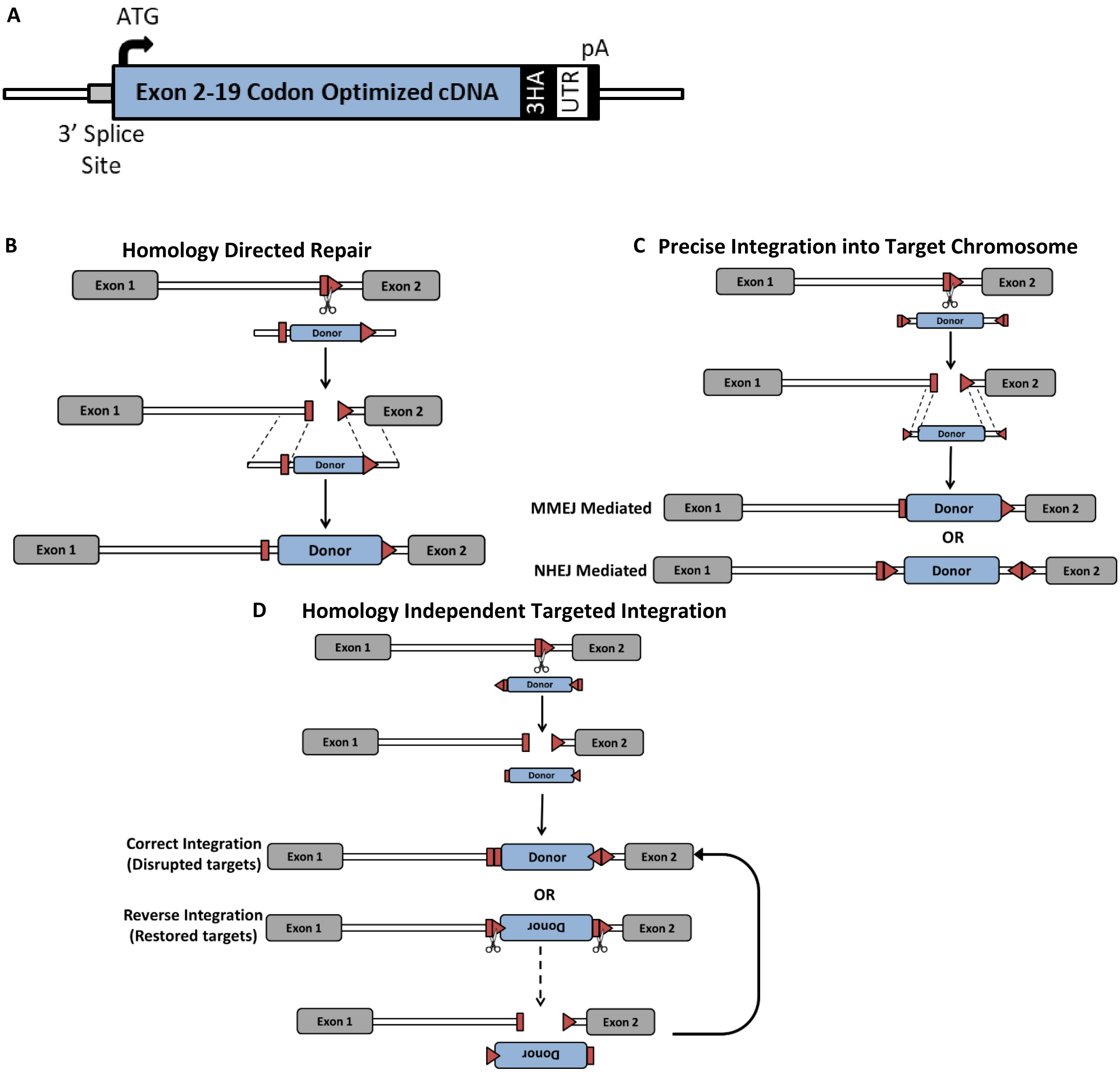

Supplemental Figure 1: Schematics of donor template and targeted integration pathways

A) Detailed diagram of an HDR donor template. Each donor has the full *BTK* coding sequence (exons 2-19) that have been codon optimized. There are 3 C terminal hemagglutinin (3HA) tags immediately after the coding sequence before the stop codon. Following the stop codon is a copy of the *BTK* 3' untranslated region (3UTR) including the poly adenylation site (pA). On the 5' end of the donor sequence is the 3' splice site from *BTK* intron 1 to allow for efficient splicing of the transgenic sequence. **B)** Targeted integration via homology directed repair (HDR). The red rectangle represents the 17 bp of the protospacer that are PAM (protospacer adjacent motif) distal, while the red triangle represents the 3 PAM proximal bp and the PAM. When both pieces are together in the correct orientation (a red arrow), there is an intact target site matching the delivered single guide RNA (represented by scissors). A cDNA donor template flanked by 500 bp of sequence homologous to the region surrounding the double stranded break can be integrated via the HDR pathway. **C)** Integration via precise integration into target chromosome (PITCh). A donor template was

generated flanked by 20 bp of microhomology matching the regions adjacent to the target DSB. Beyond the microhomology regions were two Cas9 binding sites matching the target site in the genomic DNA. As the DSB is generated in the genomic DNA, the target sites in the donor template can also be cleaved. This cleaved donor template can integrate into the genomic DNA DSB via either microhomology mediated end joining (MMEJ) or non-homologous end joining (NHEJ). MMEJ integration is expected to leave a seamless repair while NHEJ integration would produce a duplicate of the microhomology regions and potentially a portion of the donor template's Cas9 binding regions. **D)** Integration via homology independent targeted integration. A donor template with no added homology arms was flanked by one or two Cas9 binding sites that are each the reverse complement of the sequence found in the genomic DNA. Upon delivery of the reagents, both the genomic DNA and the donor template will be cleaved and the resulting cleaved donor can be integrated via NHEJ. Without homology to guide the directionality of the integration, both the in frame and reverse oriented integration events are possible. However, if the donor is integrated in reverse, the Cas9 binding sites will be restored, barring insertions or deletions (indels) at the junction. The integrated donor may then be excised until it orients in the forward orientation or indels form at either junction, preventing re-cleavage.

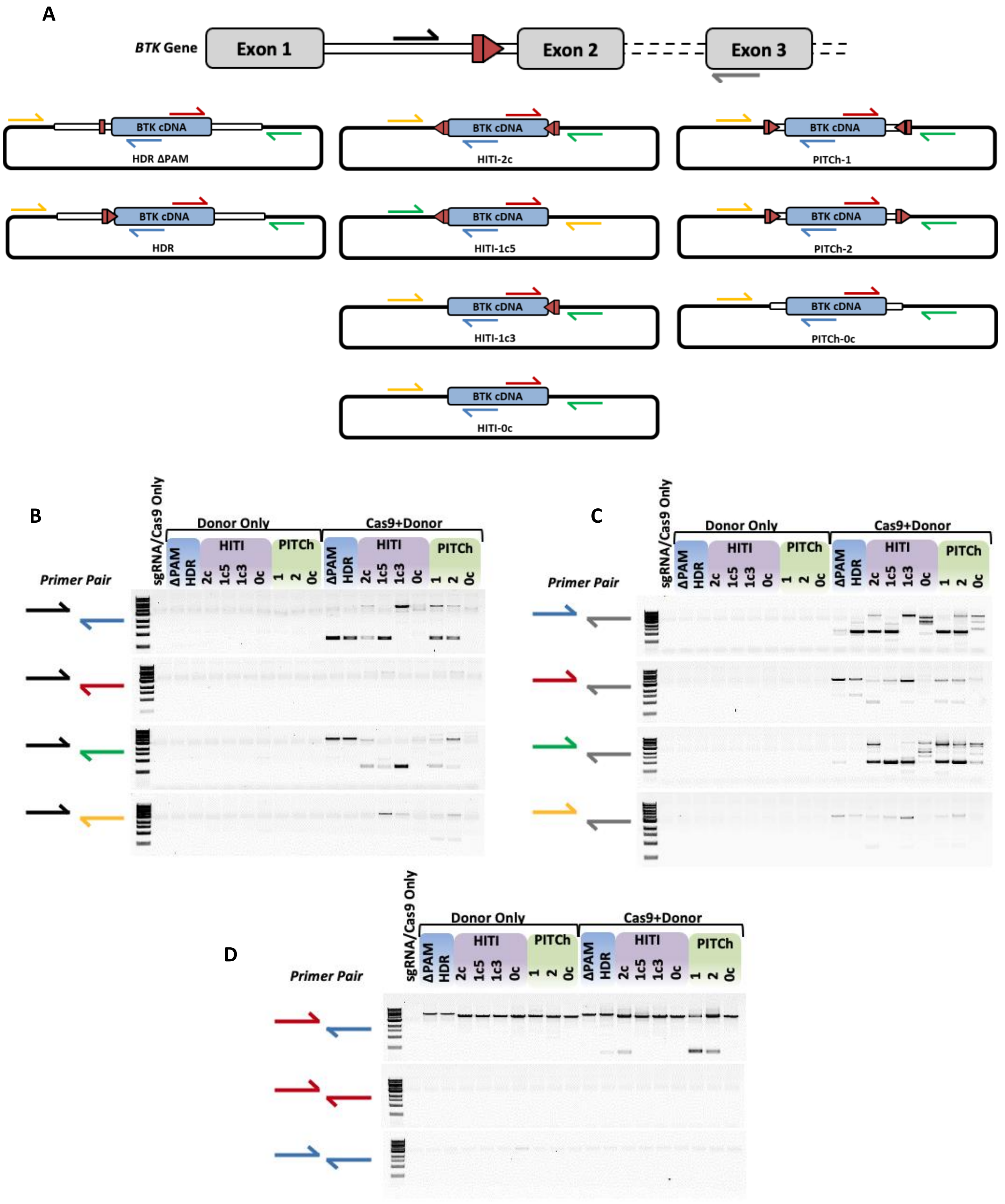
A
BTK Gene
Exon 1
Exon 2
Exon 3
BTK cDNA
HDR ΔPAM
HDR
HITI-2c
HITI-1c5
HITI-1c3
HITI-0c
PITCh-1
PITCh-2
PITCh-0c
B
C
D
Primer Pair
sgRNA/Cas9 Only
Donor Only
Cas9+Donor
HITI
PITCh
ΔPAM
HDR
2c
1c5
1c3
0c
1
2
0c

Supplemental Figure 2: Qualitative evaluation of 5’ integration junctions in K562 cells treated with HDR, HITI, or PITCh editing reagents.

A) Schematic showing primers (arrows) used for In/Out PCR of the 5’ and 3’ integration junctions and their respective binding sites on each donor template. The black primer is specific to the negative strand of *BTK* intron 1 genomic DNA while the gray primer binds to the positive strand in exon 3 just beyond the homology arm to preclude binding of unintegrated donor template. These primers were used as the “Out” primer for each of the In/Out PCRs. The blue primer binds to the to the positive strand of the 5’ end of the codon optimized *BTK* cDNA donor sequence, and is the same primer used in droplet digital PCR assays. The red primer binds the negative strand of the 3’ end of the codon optimized donor sequence. The green and yellow primers bind to the TOPO 2.1 plasmid backbone on either side of the donor cassette. Due to the flexible directionality of the TOPO TA cloning process used to assemble these donor templates, the HITI 1c5 donor cassette is oriented opposite the other donors in its backbone, meaning the relative positions of the green and yellow primer binding sites are flipped for that donor template. Genomic DNA from K562 cells treated with either sgRNA/Cas9 expression plasmid alone, one of the donor template plasmids alone, or both together was amplified to detect different integration events at the **B)** 5’ and **C)** 3’ junctions. The different primer pairs detect integration of the cDNA in either forward or reverse orientation or integration of the plasmid backbone in either orientation. **D)** The red and blue primer pairs were also used in different combinations to detect integration of donor concatamers. 10-50 ng of genomic DNA was used in the reactions above with 0.5 uM of each primer and 30-35 PCR cycles based on manufacturer’s protocol for Platinum SuperFi II Green PCR Master Mix (ThermoFisher Scientific; Waltham, MA).

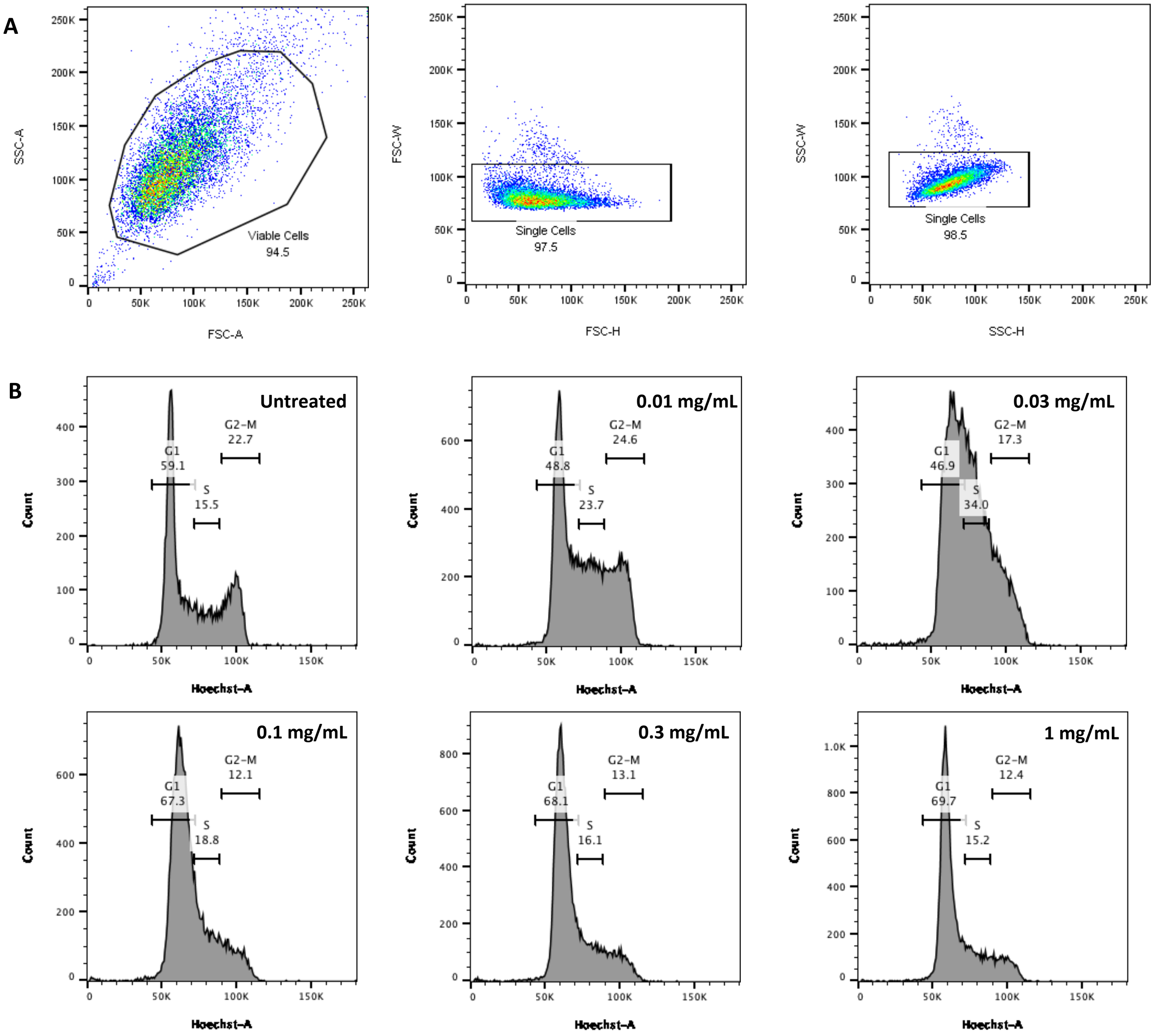

Supplemental Figure 3: Hydroxyurea cell cycle synchronization efficacy in K562 cells

A) Representative gating scheme for viability and single cells during cell cycle analysis. **B)** Hoechst dye analysis of cell cycle performed on K562 cells after incubation with increasing doses of hydroxyurea (HU).

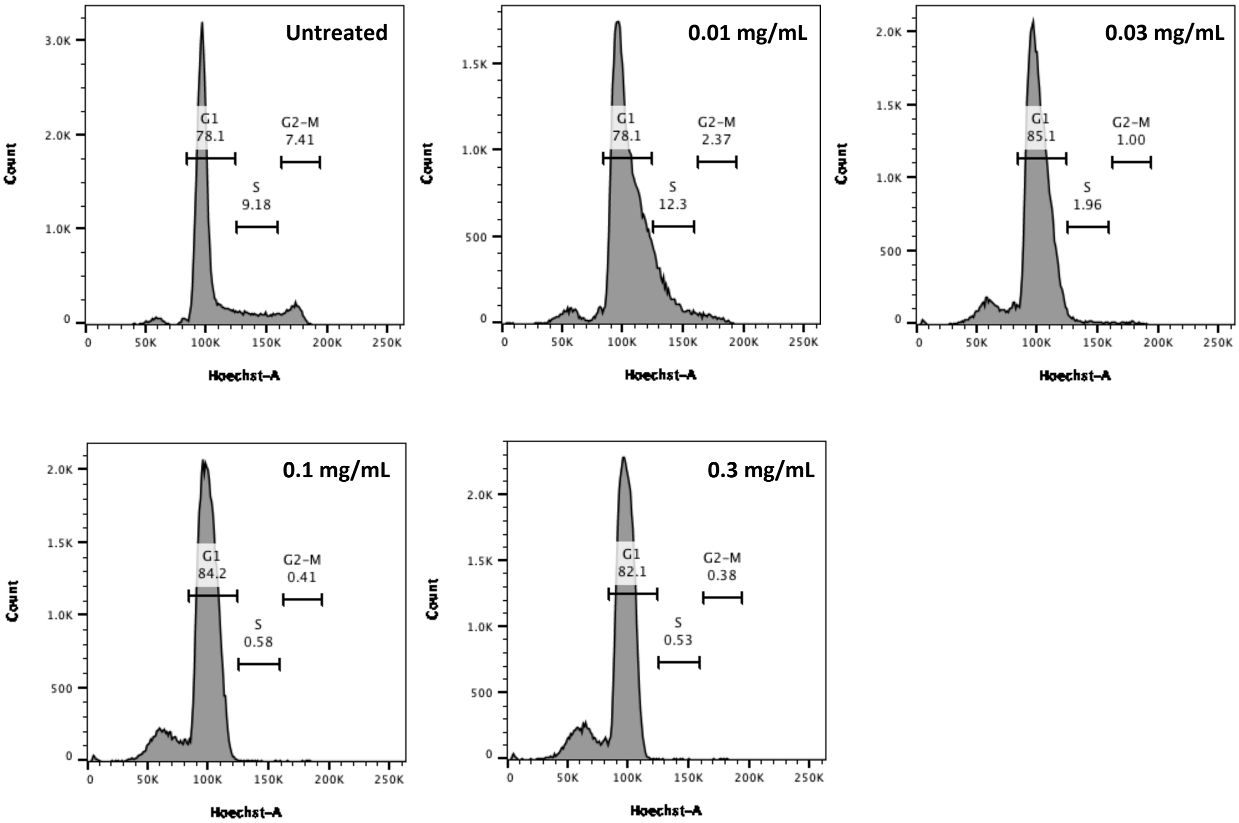

Supplemental Figure 4: Hydroxyurea cell cycle synchronization in PBSC

Hoechst dye analysis of cell cycle performed on PBSC after incubation with increasing doses of hydroxyurea (HU).

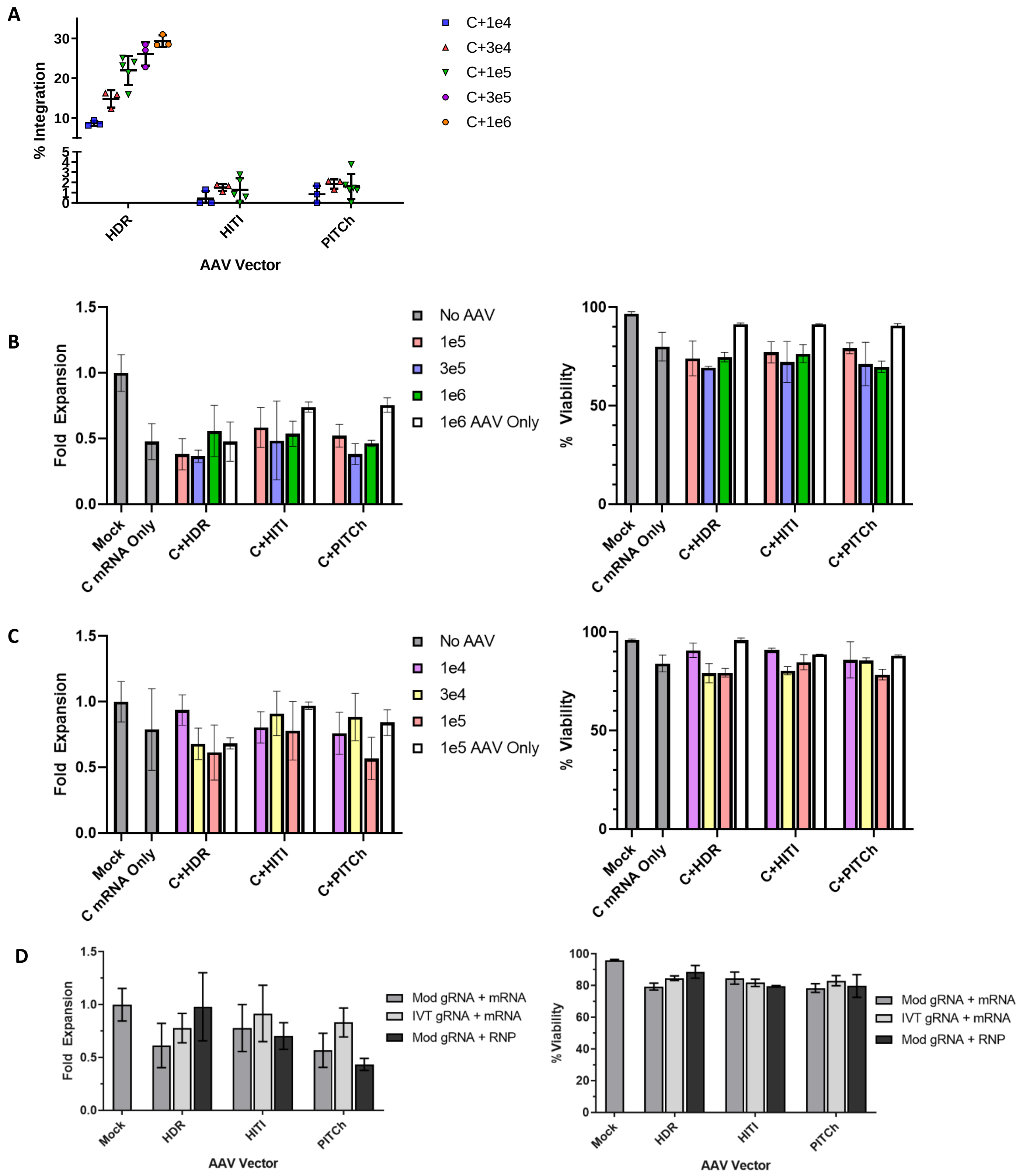

Supplemental Figure 5: Optimizing reagents for delivery into human CD34+ mobilized peripheral blood stem and progenitor cells

A) Human CD34+ cells taken from Granulocyte colony-stimulating factor mobilized peripheral blood were electroporated with Cas9 mRNA and single guide RNA (sgRNA) targeting BTK intron 1 before transduction with donor template adeno-associated viral vectors (AAVs) at various multiplicities of infections (MOIs). Data from two experiments combined (higher MOIs vs lower MOIs). **B)** Viability and fold expansion measured 1-day post electroporation. Donor templates AAVs were delivered at higher MOIs of 1e5, 3e5, or 1e6. **C)** Viability and fold expansion measured 1-day post electroporation. Donor templates AAVs were delivered at lower MOIs of 1e4, 3e4, or 1e5. **D)** Viability and fold expansion measured 1-day post electroporation from Fig 4A. Human PBSCs were treated with either *in vitro transcribed* (IVT) gRNA or chemically synthesized and modified (2'-O-methyl analogs and 3' phosphorothioate internucleotide linkages at each of the three 5' and 3' terminal RNA residues) gRNA. Cas9 was delivered either as mRNA or as RNP precomplexed with the respective gRNA. Cells were then transduced with donor AAV at an MOI of 1e5.

HDR Donor

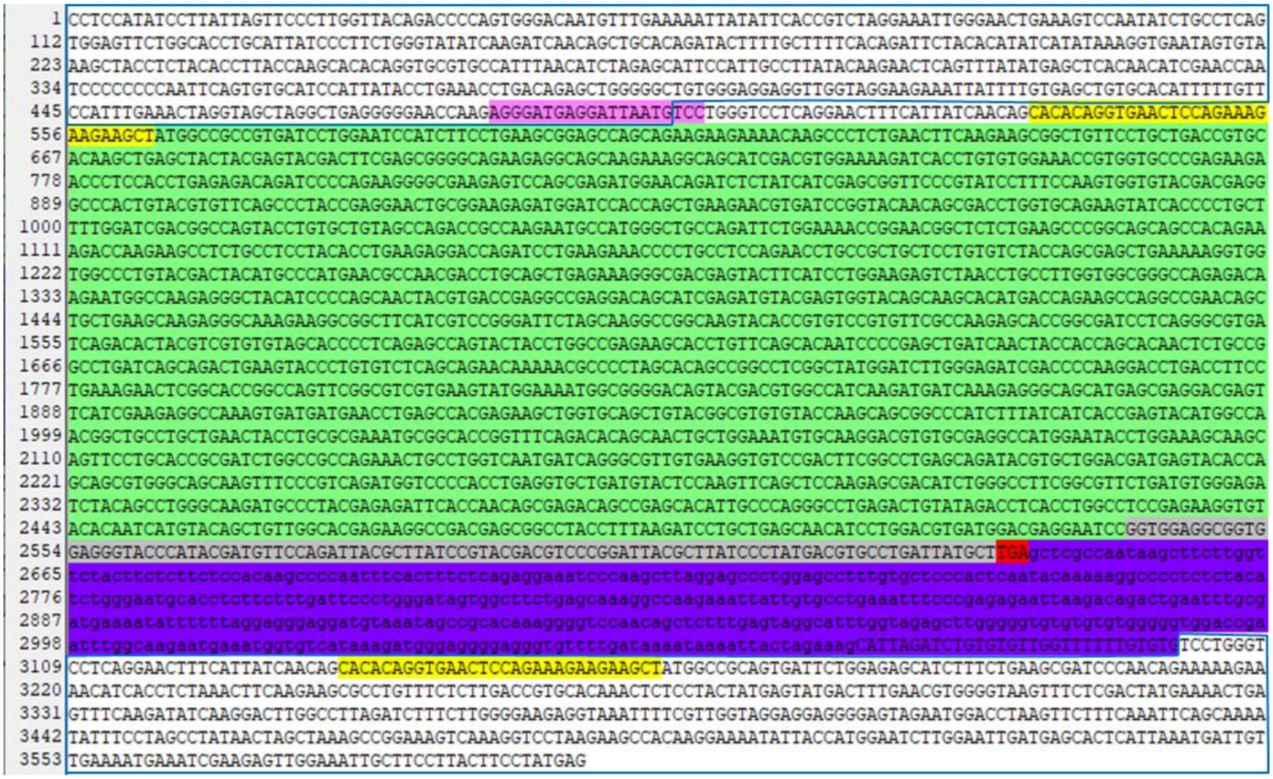

HDR ΔPAM Donor

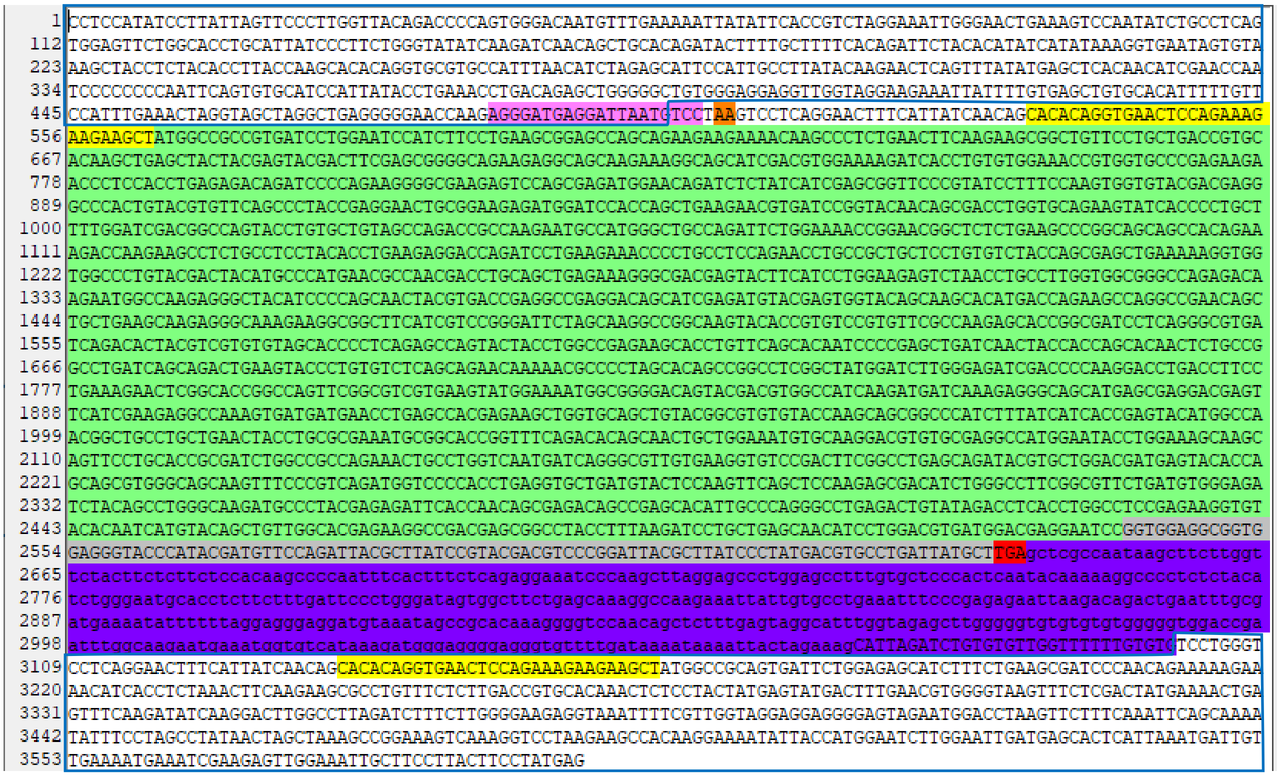

HITI-2c Donor

```
1    CCAGGACATTAATCCTCATCCCTTCCTCAGGAACTTTCATTATCAACAGCACACAGGTGAACTCCAGAAAGAAGAAGCTATGGCCGCCGTGATCCTGGAATCCATCTTCCT
112  GAAGCGGAGCCAGCAGAAGAAGAAAACAAGCCCTCTGAACTTCAAGAAGCGGCTGTTCCTGCTGACCGTGCACAAGCTGAGCTACTACGAGTACGACTTCGAGCGGGGCAG
223  AAGAGGCAGCAAGAAAGGCAGCATCGACGTGGAAAAGATCACCTGTGTGGAAACCGTGGTGCCCGAGAAGAACCCTCCACCTGAGAGACAGATCCCCAGAAGGGGCGAAGA
334  GTCCAGCGAGATGGAACAGATCTCTATCATCGAGCGGTTCCCGTATCCTTTCCAAGTGGTGTACGACGAGGGCCCACTGTACGTGTTCAGCCCTACCGAGGAACTGCGGAA
445  GAGATGGATCCACCAGCTGAAGAACGTGATCCGGTACAACAGCGACCTGGTGCAGAAGTATCACCCCTGCTTTTGGATCGACGGCCAGTACCTGTGCTGTAGCCAGACCGC
556  CAAGAATGCCATGGGCTGCCAGATTCTGGAAAACCGGAACGGCTCTCTGAAGCCCGGCAGCAGCCACAGAAAGACCAAGAAGCCTCTGCCTCCTACACCTGAAGAGGACCA
667  GATCCTGAAGAAACCCCTGCCTCCAGAACCTGCCGCTGCTCCTGTGTCTACCAGCGAGCTGAAAAAGGTGGTGGCCCTGTACGACTACATGCCCATGAACGCCAACGACCT
778  GCAGCTGAGAAAGGGCGACGAGTACTTCATCCTGGAAGAGTCTAACCTGCCTTGGTGGCGGGCCAGAGACAAGAATGGCCAAGAGGGCTACATCCCCAGCAACTACGTGAC
889  CGAGGCCGAGGACAGCATCGAGATGTACGAGTGGTACAGCAAGCACATGACCAGAAGCCAGGCCGAACAGCTGCTGAAGCAAGAGGGCAAAGAAGGCGGCTTCATCGTCCG
1000 GGATTCTAGCAAGGCCGGCAAGTACACCGTGTCCGTGTTCGCCAAGAGCACCGGCGATCCTCAGGGCGTGATCAGACACTACGTCGTGTGTAGCACCCCTCAGAGCCAGTA
1111 CTACCTGGCCGAGAAGCACCTGTTCAGCACAATCCCCGAGCTGATCAACTACCACCAGCACAACTCTGCCGGCCTGATCAGCAGACTGAAGTACCCTGTGTCTCAGCAGAA
1222 CAAAAACGCCCCTAGCACAGCCGGCCTCGGCTATGGATCTTGGGAGATCGACCCCAAGGACCTGACCTTCCTGAAAGAACTCGGCACCGGCCAGTTCGGCGTCGTGAAGTA
1333 TGGAAAATGGCGGGGACAGTACGACGTGGCCATCAAGATGATCAAAGAGGGCAGCATGAGCGAGGACGAGTTCATCGAAGAGGCCAAAGTGATGATGAACCTGAGCCACGA
1444 GAAGCTGGTGCAGCTGTACGGCGTGTGTACCAAGCAGCGGCCCATCTTTATCATCACCGAGTACATGGCCAACGGCTGCCTGCTGAACTACCTGCGCGAAATGCGGCACCG
1555 GTTTCAGACACAGCAACTGCTGGAAATGTGCAAGGACGTGTGCGAGGCCATGGAATACCTGGAAAGCAAGCAGTTCCTGCACCGCGATCTGGCCGCCAGAAACTGCCTGGT
1666 CAATGATCAGGGCGTTGTGAAGGTGTCCGACTTCGGCCTGAGCAGATACGTGCTGGACGATGAGTACACCAGCAGCGTGGGCAGCAAGTTTCCCGTCAGATGGTCCCCACC
1777 TGAGGTGCTGATGTACTCCAAGTTCAGCTCCAAGAGCGACATCTGGGCCTTCGGCGTTCTGATGTGGGAGATCTACAGCCTGGGCAAGATGCCCTACGAGAGATTCACCAA
1888 CAGCGAGACAGCCGAGCACATTGCCCAGGGCCTGAGACTGTATAGACCTCACCTGGCCTCCGAGAAGGTGTACACAATCATGTACAGCTGTTGGCACGAGAAGGCCGACGA
1999 GCGGCCTACCTTTAAGATCCTGCTGAGCAACATCCTGGACGTGATGGACGAGGAATCCGGTGGAGGCGGTGGAGGGTACCCATACGATGTTCCAGATTACGCTTATCCGTA
2110 CGACGTCCCGGATTACGCTTATCCCTATGACGTGCCTGATTATGCTTGAgctcgccaataagcttcttggttctacttctcttctccacaagccccaatttcactttctca
2221 gaggaaatcccaagcttaggagccctggagcctttgtgctcccactcaatacaaaaaggcccctctctacatctgggaatgcacctcttctttgattccctgggatagtgg
2332 cttctgagcaaaggccaagaaattattgtgcctgaaatttcccgagagaattaagacagactgaatttgcgatgaaaatatttttttaggagggaggatgtaaatagccgca
2443 caaaggggtccaacagctctttgagtaggcatttggtagagcttgggggtgtgtgtgtgggggtggaccgaatttggcaagaatgaaatggtgtcataaagatgggagggg
2554 agggtgttttgataaaataaaattactagaaagCATTAGATCTGTGTGTTGGTTTTTTGTGTGCCAGGACATTAATCCTCATCCCT
```

HITI-1c5’ Donor

```
1    CCAGGACATTAATCCTCATCCCTTCCTCAGGAACTTTCATTATCAACAGCACACAGGTGAACTCCAGAAAGAAGAAGCTATGGCCGCCGTGATCCTGGAATCCATCTTCCTG
113  AAGCGGAGCCAGCAGAAGAAGAAAACAAGCCCTCTGAACTTCAAGAAGCGGCTGTTCCTGCTGACCGTGCACAAGCTGAGCTACTACGAGTACGACTTCGAGCGGGGCAGAA
225  GAGGCAGCAAGAAAGGCAGCATCGACGTGGAAAAGATCACCTGTGTGGAAACCGTGGTGCCCGAGAAGAACCCTCCACCTGAGAGACAGATCCCCAGAAGGGGCGAAGAGTC
337  CAGCGAGATGGAACAGATCTCTATCATCGAGCGGTTCCCGTATCCTTTCCAAGTGGTGTACGACGAGGGCCCACTGTACGTGTTCAGCCCTACCGAGGAACTGCGGAAGAGA
449  TGGATCCACCAGCTGAAGAACGTGATCCGGTACAACAGCGACCTGGTGCAGAAGTATCACCCCTGCTTTTGGATCGACGGCCAGTACCTGTGCTGTAGCCAGACCGCCAAGA
561  ATGCCATGGGCTGCCAGATTCTGGAAAACCGGAACGGCTCTCTGAAGCCCGGCAGCAGCCACAGAAAGACCAAGAAGCCTCTGCCTCCTACACCTGAAGAGGACCAGATCCT
673  GAAGAAACCCCTGCCTCCAGAACCTGCCGCTGCTCCTGTGTCTACCAGCGAGCTGAAAAAGGTGGTGGCCCTGTACGACTACATGCCCATGAACGCCAACGACCTGCAGCTG
785  AGAAAGGGCGACGAGTACTTCATCCTGGAAGAGTCTAACCTGCCTTGGTGGCGGGCCAGAGACAAGAATGGCCAAGAGGGCTACATCCCCAGCAACTACGTGACCGAGGCCG
897  AGGACAGCATCGAGATGTACGAGTGGTACAGCAAGCACATGACCAGAAGCCAGGCCGAACAGCTGCTGAAGCAAGAGGGCAAAGAAGGCGGCTTCATCGTCCGGGATTCTAG
1009 CAAGGCCGGCAAGTACACCGTGTCCGTGTTCGCCAAGAGCACCGGCGATCCTCAGGGCGTGATCAGACACTACGTCGTGTGTAGCACCCCTCAGAGCCAGTACTACCTGGCC
1121 GAGAAGCACCTGTTCAGCACAATCCCCGAGCTGATCAACTACCACCAGCACAACTCTGCCGGCCTGATCAGCAGACTGAAGTACCCTGTGTCTCAGCAGAACAAAAACGCCC
1233 CTAGCACAGCCGGCCTCGGCTATGGATCTTGGGAGATCGACCCCAAGGACCTGACCTTCCTGAAAGAACTCGGCACCGGCCAGTTCGGCGTCGTGAAGTATGGAAAATGGCG
1345 GGGACAGTACGACGTGGCCATCAAGATGATCAAAGAGGGCAGCATGAGCGAGGACGAGTTCATCGAAGAGGCCAAAGTGATGATGAACCTGAGCCACGAGAAGCTGGTGCAG
1457 CTGTACGGCGTGTGTACCAAGCAGCGGCCCATCTTTATCATCACCGAGTACATGGCCAACGGCTGCCTGCTGAACTACCTGCGCGAAATGCGGCACCGGTTTCAGACACAGC
1569 AACTGCTGGAAATGTGCAAGGACGTGTGCGAGGCCATGGAATACCTGGAAAGCAAGCAGTTCCTGCACCGCGATCTGGCCGCCAGAAACTGCCTGGTCAATGATCAGGGCGT
1681 TGTGAAGGTGTCCGACTTCGGCCTGAGCAGATACGTGCTGGACGATGAGTACACCAGCAGCGTGGGCAGCAAGTTTCCCGTCAGATGGTCCCCACCTGAGGTGCTGATGTAC
1793 TCCAAGTTCAGCTCCAAGAGCGACATCTGGGCCTTCGGCGTTCTGATGTGGGAGATCTACAGCCTGGGCAAGATGCCCTACGAGAGATTCACCAACAGCGAGACAGCCGAGC
1905 ACATTGCCCAGGGCCTGAGACTGTATAGACCTCACCTGGCCTCCGAGAAGGTGTACACAATCATGTACAGCTGTTGGCACGAGAAGGCCGACGAGCGGCCTACCTTTAAGAT
2017 CCTGCTGAGCAACATCCTGGACGTGATGGACGAGGAATCCGGTGGAGGCGGTGGAGGGTACCCATACGATGTTCCAGATTACGCTTATCCGTACGACGTCCCGGATTACGCT
2129 TATCCCTATGACGTGCCTGATTATGCTTGAgctcgccaataagcttcttggttctacttctcttctccacaagccccaatttcactttctcagaggaaatcccaagcttagg
2241 agccctggagcctttgtgctcccactcaatacaaaaaggcccctctctacatctgggaatgcacctcttctttgattccctgggatagtggcttctgagcaaaggccaagaa
2353 attattgtgcctgaaatttcccgagagaattaagacagactgaatttgcgatgaaaatatttttttaggagggaggatgtaaatagccgcacaaaggggtccaacagctcttt
2465 gagtaggcatttggtagagcttgggggtgtgtgtgtgggggtggaccgaatttggcaagaatgaaatggtgtcataaagatgggaggggagggtgttttgataaaataaaat
2577 tactagaaagCATTAGATCTGTGTGTTGGTTTTTTGTGTG
```

HITI-1c3’ Donor

```
1    TCCTCAGGAACTTTCATTATCAACAGCACACAGGTGAACTCCAGAAAGAAGAAGCTATGGCCGCCGTGATCCTGGAATCCATCTTCCTGAAGCGGAGCCAGCAGAAGAAGAA
113  AACAAGCCCTCTGAACTTCAAGAAGCGGCTGTTCCTGCTGACCGTGCACAAGCTGAGCTACTACGAGTACGACTTCGAGCGGGGCAGAAGAGGCAGCAAGAAAGGCAGCATC
225  GACGTGGAAAAGATCACCTGTGTGGAAACCGTGGTGCCCGAGAAGAACCCTCCACCTGAGAGACAGATCCCCAGAAGGGGCGAAGAGTCCAGCGAGATGGAACAGATCTCTA
337  TCATCGAGCGGTTCCCGTATCCTTTCCAAGTGGTGTACGACGAGGGCCCACTGTACGTGTTCAGCCCTACCGAGGAACTGCGGAAGAGATGGATCCACCAGCTGAAGAACGT
449  GATCCGGTACAACAGCGACCTGGTGCAGAAGTATCACCCCTGCTTTTGGATCGACGGCCAGTACCTGTGCTGTAGCCAGACCGCCAAGAATGCCATGGGCTGCCAGATTCTG
561  GAAAACCGGAACGGCTCTCTGAAGCCCGGCAGCAGCCACAGAAAGACCAAGAAGCCTCTGCCTCCTACACCTGAAGAGGACCAGATCCTGAAGAAACCCCTGCCTCCAGAAC
673  CTGCCGCTGCTCCTGTGTCTACCAGCGAGCTGAAAAAGGTGGTGGCCCTGTACGACTACATGCCCATGAACGCCAACGACCTGCAGCTGAGAAAGGGCGACGAGTACTTCAT
785  CCTGGAAGAGTCTAACCTGCCTTGGTGGCGGGCCAGAGACAAGAATGGCCAAGAGGGCTACATCCCCAGCAACTACGTGACCGAGGCCGAGGACAGCATCGAGATGTACGAG
897  TGGTACAGCAAGCACATGACCAGAAGCCAGGCCGAACAGCTGCTGAAGCAAGAGGGCAAAGAAGGCGGCTTCATCGTCCGGGATTCTAGCAAGGCCGGCAAGTACACCGTGT
1009 CCGTGTTCGCCAAGAGCACCGGCGATCCTCAGGGCGTGATCAGACACTACGTCGTGTGTAGCACCCCTCAGAGCCAGTACTACCTGGCCGAGAAGCACCTGTTCAGCACAAT
1121 CCCCGAGCTGATCAACTACCACCAGCACAACTCTGCCGGCCTGATCAGCAGACTGAAGTACCCTGTGTCTCAGCAGAACAAAAACGCCCCTAGCACAGCCGGCCTCGGCTAT
1233 GGATCTTGGGAGATCGACCCCAAGGACCTGACCTTCCTGAAAGAACTCGGCACCGGCCAGTTCGGCGTCGTGAAGTATGGAAAATGGCGGGGACAGTACGACGTGGCCATCA
1345 AGATGATCAAAGAGGGCAGCATGAGCGAGGACGAGTTCATCGAAGAGGCCAAAGTGATGATGAACCTGAGCCACGAGAAGCTGGTGCAGCTGTACGGCGTGTGTACCAAGCA
1457 GCGGCCCATCTTTATCATCACCGAGTACATGGCCAACGGCTGCCTGCTGAACTACCTGCGCGAAATGCGGCACCGGTTTCAGACACAGCAACTGCTGGAAATGTGCAAGGAC
1569 GTGTGCGAGGCCATGGAATACCTGGAAAGCAAGCAGTTCCTGCACCGCGATCTGGCCGCCAGAAACTGCCTGGTCAATGATCAGGGCGTTGTGAAGGTGTCCGACTTCGGCC
1681 TGAGCAGATACGTGCTGGACGATGAGTACACCAGCAGCGTGGGCAGCAAGTTTCCCGTCAGATGGTCCCCACCTGAGGTGCTGATGTACTCCAAGTTCAGCTCCAAGAGCGA
1793 CATCTGGGCCTTCGGCGTTCTGATGTGGGAGATCTACAGCCTGGGCAAGATGCCCTACGAGAGATTCACCAACAGCGAGACAGCCGAGCACATTGCCCAGGGCCTGAGACTG
1905 TATAGACCTCACCTGGCCTCCGAGAAGGTGTACACAATCATGTACAGCTGTTGGCACGAGAAGGCCGACGAGCGGCCTACCTTTAAGATCCTGCTGAGCAACATCCTGGACG
2017 TGATGGACGAGGAATCCGGTGGAGGCGGTGGAGGGTACCCATACGATGTTCCAGATTACGCTTATCCGTACGACGTCCCGGATTACGCTTATCCCTATGACGTGCCTGATTA
2129 TGCTTGAgctcgccaataagcttcttggttctacttctcttctccacaagccccaatttcactttctcagaggaaatcccaagcttaggagccctggagcctttgtgctccc
2241 actcaatacaaaaaggcccctctctacatctgggaatgcacctcttctttgattccctgggatagtggcttctgagcaaaggccaagaaattattgtgcctgaaatttcccg
2353 agagaattaagacagactgaatttgcgatgaaaatatttttttaggagggaggatgtaaatagccgcacaaaggggtccaacagctctttgagtaggcatttggtagagcttg
2465 ggggtgtgtgtgtgggggtggaccgaatttggcaagaatgaaatggtgtcataaagatgggaggggagggtgttttgataaaataaaattactagaaagCATTAGATCTGTG
2577 TGTTGGTTTTTGTGTGCCAGGACATTAATCCTCATCCCT
```

HITI-0c Donor

```
1    TCCTCAGGAACTTTCATTATCAACAGCACACAGGTGAACTCCAGAAAGAAGAAGCTATGGCCGCCGTGATCCTGGAATCCATCTTCCTGAAGCGGAGCCAGCAGAAGAAGAA
113  AACAAGCCCTCTGAACTTCAAGAAGCGGCTGTTCCTGCTGACCGTGCACAAGCTGAGCTACTACGAGTACGACTTCGAGCGGGGCAGAAGAGGCAGCAAGAAAGGCAGCATC
225  GACGTGGAAAAGATCACCTGTGTGGAAACCGTGGTGCCCGAGAAGAACCCTCCACCTGAGAGACAGATCCCCAGAAGGGGCGAAGAGTCCAGCGAGATGGAACAGATCTCTA
337  TCATCGAGCGGTTCCCGTATCCTTTCCAAGTGGTGTACGACGAGGGCCCACTGTACGTGTTCAGCCCTACCGAGGAACTGCGGAAGAGATGGATCCACCAGCTGAAGAACGT
449  GATCCGGTACAACAGCGACCTGGTGCAGAAGTATCACCCCTGCTTTTGGATCGACGGCCAGTACCTGTGCTGTAGCCAGACCGCCAAGAATGCCATGGGCTGCCAGATTCTG
561  GAAAACCGGAACGGCTCTCTGAAGCCCGGCAGCAGCCACAGAAAGACCAAGAAGCCTCTGCCTCCTACACCTGAAGAGGACCAGATCCTGAAGAAACCCCTGCCTCCAGAAC
673  CTGCCGCTGCTCCTGTGTCTACCAGCGAGCTGAAAAAGGTGGTGGCCCTGTACGACTACATGCCCATGAACGCCAACGACCTGCAGCTGAGAAAGGGCGACGAGTACTTCAT
785  CCTGGAAGAGTCTAACCTGCCTTGGTGGCGGGCCAGAGACAAGAATGGCCAAGAGGGCTACATCCCCAGCAACTACGTGACCGAGGCCGAGGACAGCATCGAGATGTACGAG
897  TGGTACAGCAAGCACATGACCAGAAGCCAGGCCGAACAGCTGCTGAAGCAAGAGGGCAAAGAAGGCGGCTTCATCGTCCGGGATTCTAGCAAGGCCGGCAAGTACACCGTGT
1009 CCGTGTTCGCCAAGAGCACCGGCGATCCTCAGGGCGTGATCAGACACTACGTCGTGTGTAGCACCCCTCAGAGCCAGTACTACCTGGCCGAGAAGCACCTGTTCAGCACAAT
1121 CCCCGAGCTGATCAACTACCACCAGCACAACTCTGCCGGCCTGATCAGCAGACTGAAGTACCCTGTGTCTCAGCAGAACAAAAACGCCCCTAGCACAGCCGGCCTCGGCTAT
1233 GGATCTTGGGAGATCGACCCCAAGGACCTGACCTTCCTGAAAGAACTCGGCACCGGCCAGTTCGGCGTCGTGAAGTATGGAAAATGGCGGGGACAGTACGACGTGGCCATCA
1345 AGATGATCAAAGAGGGCAGCATGAGCGAGGACGAGTTCATCGAAGAGGCCAAAGTGATGATGAACCTGAGCCACGAGAAGCTGGTGCAGCTGTACGGCGTGTGTACCAAGCA
1457 GCGGCCCATCTTTATCATCACCGAGTACATGGCCAACGGCTGCCTGCTGAACTACCTGCGCGAAATGCGGCACCGGTTTCAGACACAGCAACTGCTGGAAATGTGCAAGGAC
1569 GTGTGCGAGGCCATGGAATACCTGGAAAGCAAGCAGTTCCTGCACCGCGATCTGGCCGCCAGAAACTGCCTGGTCAATGATCAGGGCGTTGTGAAGGTGTCCGACTTCGGCC
1681 TGAGCAGATACGTGCTGGACGATGAGTACACCAGCAGCGTGGGCAGCAAGTTTCCCGTCAGATGGTCCCCACCTGAGGTGCTGATGTACTCCAAGTTCAGCTCCAAGAGCGA
1793 CATCTGGGCCTTCGGCGTTCTGATGTGGGAGATCTACAGCCTGGGCAAGATGCCCTACGAGAGATTCACCAACAGCGAGACAGCCGAGCACATTGCCCAGGGCCTGAGACTG
1905 TATAGACCTCACCTGGCCTCCGAGAAGGTGTACACAATCATGTACAGCTGTTGGCACGAGAAGGCCGACGAGCGGCCTACCTTTAAGATCCTGCTGAGCAACATCCTGGACG
2017 TGATGGACGAGGAATCCGGTGGAGGCGGTGGAGGGTACCCATACGATGTTCCAGATTACGCTTATCCGTACGACGTCCCGGATTACGCTTATCCCTATGACGTGCCTGATTA
2129 TGCTTGAgctcgccaataagcttcttggttctacttctcttctccacaagccccaatttcactttctcagaggaaatcccaagcttaggagccctggagcctttgtgctccc
2241 actcaatacaaaaaggcccctctctacatctgggaatgcacctcttctttgattccctgggatagtggcttctgagcaaaggccaagaaattattgtgcctgaaatttcccg
2353 agagaattaagacagactgaatttgcgatgaaaatatttttttaggagggaggatgtaaatagccgcacaaaggggtccaacagctctttgagtaggcatttggtagagcttg
2465 ggggtgtgtgtgtgggggtggaccgaatttggcaagaatgaaatggtgtcataaagatgggaggggagggtgttttgataaaataaaattactagaaagCATTAGATCTGTG
2577 TGTTGGTTTTTGTGTG
```

PITCh-1 Donor

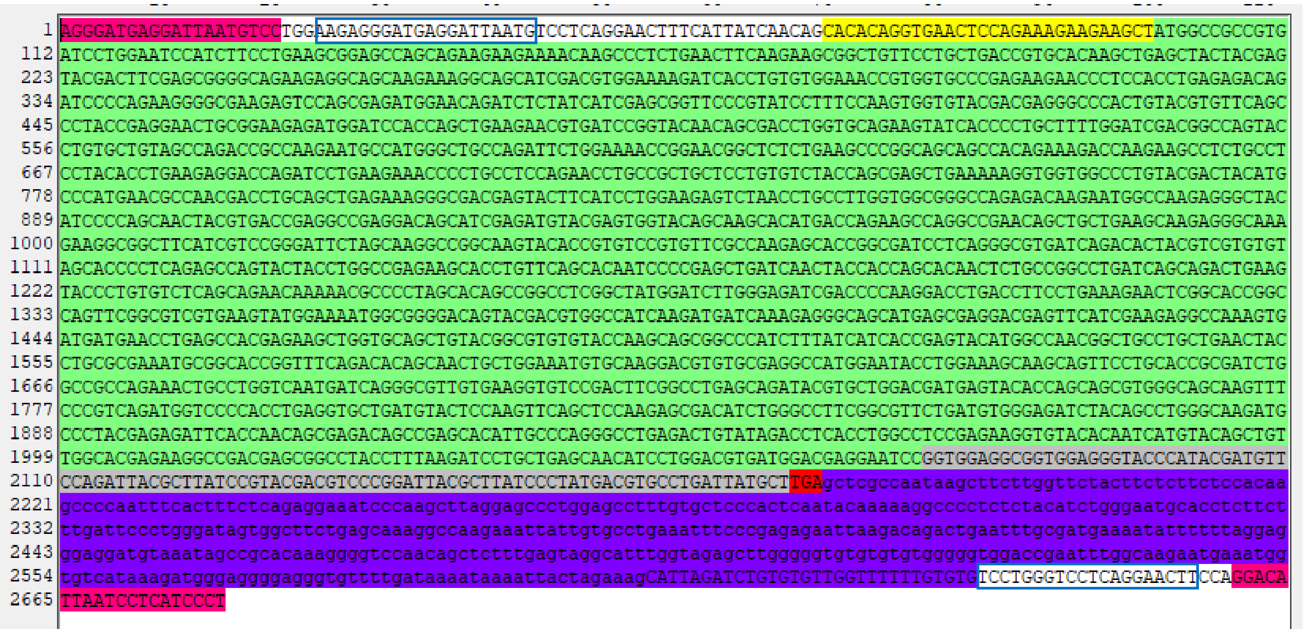

PITCh-2 Donor

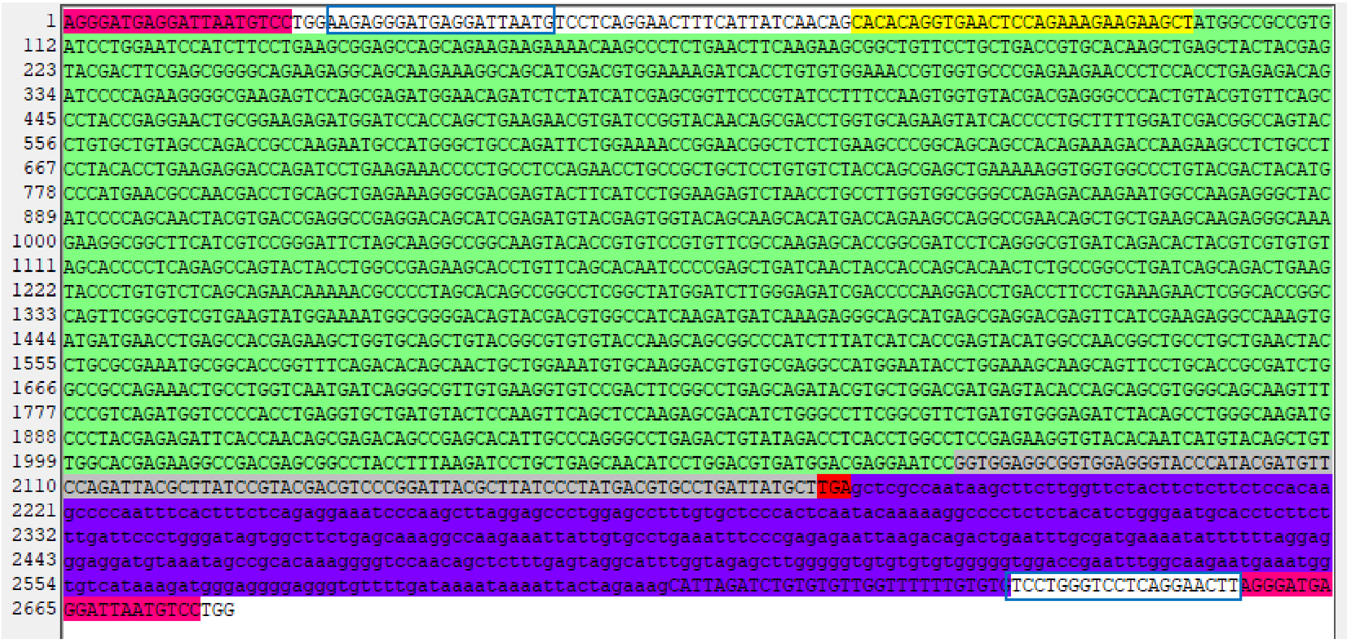

PITCH-0c Donor

```
   1 AAGAGGGATGAGGATTAATGTCCTCAGGAACTTTCATTATCAACAGCACACAGGTGAACTCCAGAAAGAAGAAGCTATGGCCGCCGTGATCCTGGAATCCATCTTCCTGAA
 112 GCGGAGCCAGCAGAAGAAGAAAACAAGCCCTCTGAACTTCAAGAAGCGGCTGTTCCTGCTGACCGTGCACAAGCTGAGCTACTACGAGTACGACTTCGAGCGGGGCAGAAG
 223 AGGCAGCAAGAAAGGCAGCATCGACGTGGAAAAGATCACCTGTGTGGAAACCGTGGTGCCCGAGAAGAACCCTCCACCTGAGAGACAGATCCCCAGAAGGGGCGAAGAGTC
 334 CAGCGAGATGGAACAGATCTCTATCATCGAGCGGTTCCCGTATCCTTTCCAAGTGGTGTACGACGAGGGCCCACTGTACGTGTTCAGCCCTACCGAGGAACTGCGGAAGAG
 445 ATGGATCCACCAGCTGAAGAACGTGATCCGGTACAACAGCGACCTGGTGCAGAAGTATCACCCCTGCTTTTGGATCGACGGCCAGTACCTGTGCTGTAGCCAGACCGCCAA
 556 GAATGCCATGGGCTGCCAGATTCTGGAAAACCGGAACGGCTCTCTGAAGCCCGGCAGCAGCCACAGAAAGACCAAGAAGCCTCTGCCTCCTACACCTGAAGAGGACCAGAT
 667 CCTGAAGAAACCCCTGCCTCCAGAACCTGCCGCTGCTCCTGTGTCTACCAGCGAGCTGAAAAAGGTGGTGGCCCTGTACGACTACATGCCCATGAACGCCAACGACCTGCA
 778 GCTGAGAAAGGGCGACGAGTACTTCATCCTGGAAGAGTCTAACCTGCCTTGGTGGCGGGCCAGAGACAAGAATGGCCAAGAGGGCTACATCCCCAGCAACTACGTGACCGA
 889 GGCCGAGGACAGCATCGAGATGTACGAGTGGTACAGCAAGCACATGACCAGAAGCCAGGCCGAACAGCTGCTGAAGCAAGAGGGCAAAGAAGGCGGCTTCATCGTCCGGGA
1000 TTCTAGCAAGGCCGGCAAGTACACCGTGTCCGTGTTCGCCAAGAGCACCGGCGATCCTCAGGGCGTGATCAGACACTACGTCGTGTGTAGCACCCCTCAGAGCCAGTACTA
1111 CCTGGCCGAGAAGCACCTGTTCAGCACAATCCCCGAGCTGATCAACTACCACCAGCACAACTCTGCCGGCCTGATCAGCAGACTGAAGTACCCTGTGTCTCAGCAGAACAA
1222 AAACGCCCCTAGCACAGCCGGCCTCGGCTATGGATCTTGGGAGATCGACCCCAAGGACCTGACCTTCCTGAAAGAACTCGGCACCGGCCAGTTCGGCGTCGTGAAGTATGG
1333 AAAATGGCGGGGACAGTACGACGTGGCCATCAAGATGATCAAAGAGGGCAGCATGAGCGAGGACGAGTTCATCGAAGAGGCCAAAGTGATGATGAACCTGAGCCACGAGAA
1444 GCTGGTGCAGCTGTACGGCGTGTGTACCAAGCAGCGGCCCATCTTTATCATCACCGAGTACATGGCCAACGGCTGCCTGCTGAACTACCTGCGCGAAATGCGGCACCGGTT
1555 TCAGACACAGCAACTGCTGGAAATGTGCAAGGACGTGTGCGAGGCCATGGAATACCTGGAAAGCAAGCAGTTCCTGCACCGCGATCTGGCCGCCAGAAACTGCCTGGTCAA
1666 TGATCAGGGCGTTGTGAAGGTGTCCGACTTCGGCCTGAGCAGATACGTGCTGGACGATGAGTACACCAGCAGCGTGGGCAGCAAGTTTCCCGTCAGATGGTCCCCACCTGA
1777 GGTGCTGATGTACTCCAAGTTCAGCTCCAAGAGCGACATCTGGGCCTTCGGCGTTCTGATGTGGGAGATCTACAGCCTGGGCAAGATGCCCTACGAGAGATTCACCAACAG
1888 CGAGACAGCCGAGCACATTGCCCAGGGCCTGAGACTGTATAGACCTCACCTGGCCTCCGAGAAGGTGTACACAATCATGTACAGCTGTTGGCACGAGAAGGCCGACGAGCG
1999 GCCTACCTTTAAGATCCTGCTGAGCAACATCCTGGACGTGATGGACGAGGAATCCGGTGGAGGCGGTGGAGGGTACCCATACGATGTTCCAGATTACGCTTATCCGTACGA
2110 CGTCCCGGATTACGCTTATCCCTATGACGTGCCTGATTATGCTTGAgctcgccaataagcttcttggttctacttctcttctccacaagccccaatttcactttctcagag
2221 gaaatcccaagcttaggagccctggagcctttgtgctcccactcaatacaaaaaggcccctctctacatctgggaatgcacctcttctttgattccctgggatagtggctt
2332 ctgagcaaaggccaagaaattattgtgcctgaaatttcccgagagaattaagacagactgaatttgcgatgaaaatattttttaggagggaggatgtaaatagccgcacaa
2443 aggggtccaacagctctttgagtaggcatttggtagagcttgggggtgtgtgtgtgggggtggaccgaatttggcaagaatgaaatggtgtcataaagatgggaggggagg
2554 gtgttttgataaaataaaattactagaaagCATTAGATCTGTGTGTTGGTTTTTTGTGTGTCCTGGGTCCTCAGGAACTT
```

Supplemental Figure 6: Annotated schematics of each of the nine donors that were cloned into the TOPO-2.1 plasmid backbone or an Adeno Associated Viral vector packaging plasmid backbone. The protospacer sequence is represented by pink. Homology arms are enclosed in a blue box. Yellow highlighting represents the non-coding portion of BTK exon 2. The coding regions of the BTK cDNA (exons 2-19) are in green. The 3 C terminal hemagglutinin tags and linker sequence are in gray, immediately preceding the stop codon in red. The 3' UTR sequence is in purple.
