# Supplementary material for: A comparison of DNA repair pathways to achieve a site-specific gene modification of the Bruton's tyrosine kinase gene
Source: Mol Ther Nucleic Acids. 2021 Dec 14;27:505–16. doi: 10.1016/j.omtn.2021.12.014 (PMC8728535; doi:10.1016/j.omtn.2021.12.014)
Supplement: Document S2. Article plus supplemental information [file mmc2.pdf]

Molecular Therapy
Nucleic Acids
Original Article

A comparison of DNA repair pathways to achieve a site-specific gene modification of the Bruton's tyrosine kinase gene

David H. Gray,[1] Jasmine Santos,[2] Alexandra Grace Keir,[2] Isaac Villegas,[3] Simon Maddock,[2] Edward C. Trope,[3] Joseph D. Long,[3] and Caroline Y. Kuo[3]

[1]Molecular Biology Interdepartmental Graduate Program, University of California, Los Angeles, CA 90095, USA; [2]Department of Microbiology, Immunology & Molecular Genetics, University of California, Los Angeles, CA 90095, USA; [3]Division of Allergy and Immunology, Department of Pediatrics, David Geffen School of Medicine at the University of California, 10833 Le Conte MDCC 12-430, Los Angeles, CA 90095, USA

Gene editing utilizing homology-directed repair has advanced significantly for many monogenic diseases of the hematopoietic system in recent years but has also been hindered by decreases between *in vitro* and *in vivo* gene integration rates. Homology-directed repair occurs primarily in the S/G_2 phases of the cell cycle, whereas long-term engrafting hematopoietic stem cells are typically quiescent. Alternative methods for a targeted integration have been proposed including homology-independent targeted integration and precise integration into target chromosome, which utilize non-homologous end joining and microhomology-mediated end joining, respectively. Non-homologous end joining occurs throughout the cell cycle, while microhomology-mediated end joining occurs predominantly in the S phase. We compared these pathways for the integration of a corrective DNA cassette at the Bruton's tyrosine kinase gene for the treatment of X-linked agammaglobulinemia. Homology-directed repair generated the most integration in K562 cells; however, synchronizing cells into G_1 resulted in the highest integration rates with homology-independent targeted integration. Only homology-directed repair produced seamless junctions, making it optimal for targets where insertions and deletions are impermissible. Bulk CD34+ cells were best edited by homology-directed repair and precise integration into the target chromosome, while sorted hematopoietic stem cells contained similar integration rates using all corrective donors.

INTRODUCTION

The identification of targeted endonucleases, particularly the RNA-guided clustered regularly interspaced short palindromic repeats (CRISPR)-associated proteins, has brought genome editing to the forefront in the field of gene therapy.[1,2] These endonucleases can create double-stranded breaks (DSBs) in genomic DNA with high efficiency and specificity, initiating one of several cellular repair pathways. The most thoroughly described DSB repair pathways are non-homologous end joining (NHEJ) and homology-directed repair (HDR). NHEJ is an error-prone process that repairs the DSB while introducing random nucleotide insertions and deletions (indels) at the repair junction.[3] In contrast, HDR uses a homologous template, typically a sister chromatid during cell replication, for a seamless repair of the break.[4] Each of these two methods has been harnessed for different purposes. NHEJ is most commonly used to disrupt elements in the genome through the introduction of indels at the DSB, while HDR is used for the targeted correction of single base pairs (bp) or the insertion of large sequences of DNA for site-specific gene replacement.[5–9]

Ex vivo gene editing for diseases of the hematopoietic system, such as primary immunodeficiencies and hemoglobinopathies, is progressing toward the clinic with many promising therapeutic avenues underway. However, murine studies of HDR-based gene editing in hematopoietic stem and progenitor cells (HSPCs) by many groups have come upon a common hurdle: the rates of editing via the HDR pathway in human HSPCs often drop substantially over time in murine xenograft models, while NHEJ-mediated editing typically persists at stable rates.[10–13] This discrepancy is likely due to the cell-cycle dependency of each repair pathway. HDR is generally restricted to the S/G_2 phases of the cell cycle, while NHEJ is active throughout all phases of the cell cycle.[14] Previous work has shown that using a Cas9 variant that is degraded specifically in G_0/G_1 improved the ratio of HDR to NHEJ events.[15] However, true hematopoietic stem cells (HSCs) exist mostly in G_0/G_1 and may be inherently less capable of HDR than of actively dividing progenitor cells.[16] Targeted integration into HSCs could be enhanced by either improving the effectiveness of HDR in quiescent cells or identifying methods of integration that utilize alternative mechanisms of DNA repair. One such method to improve HDR in quiescent cells is to temporarily induce cell cycling during the editing process before returning the cells to quiescence.[17] Alternatives to

Received 13 August 2021; accepted 9 December 2021;
https://doi.org/10.1016/j.omtn.2021.12.014.

Correspondence: Caroline Y. Kuo, MD, Division of Allergy and Immunology, Department of Pediatrics, David Geffen School of Medicine at the University of California, 10833 Le Conte MDCC 12-430, Los Angeles, CA 90095, USA.
E-mail: ckuo@mednet.ucla.edu

This is an open access article under the CC BY license (http://creativecommons.org/licenses/by/4.0/).

HDR for a targeted integration into DSBs include homology-independent targeted integration (HITI) and precise integration into target chromosome (PITCh), which rely on NHEJ and microhomology-mediated end joining (MMEJ), respectively.[18,19] HITI is expected to function throughout all phases of the cell cycle, while PITCh occurs primarily in late G_1/S phases due to its reliance on MMEJ.[3,20]

In this article, each of these three methods of DNA integration—HDR, HITI, and PITCh—was assessed for their potential to achieve a permanent gene-therapy-based cure for X-linked agammaglobulinemia (XLA) through gene editing of autologous HSPCs. XLA is a primary immunodeficiency resulting from a block in the pre-B cell phase of early B-lymphocyte development, which leads to a lack of mature B-lymphocytes and of antibody production in most patients.[21–24] The current standard of care for XLA is immunoglobulin-replacement infusions with prompt treatment of infections with antimicrobials. This treatment approach is life-saving and typically leads to a substantially improved quality of life, although it remains imperfect. Even with immunoglobulin supplementation, patients can experience recurrent bacterial infections, are susceptible to life-threatening viral infections with certain organisms such as enterovirus, and have overall reduced life expectancies. Additionally, immunoglobulin replacement is expensive and requires weekly to monthly infusions for a patient's lifetime.

Patients with XLA have loss-of-function mutations in the Bruton's tyrosine kinase (*BTK*) gene, which encodes a cytoplasmic signaling protein found in most hematopoietic lineages.[22] While previous work using integrating viral vectors to deliver new copies of the *BTK* gene to patient HSPCs has produced promising results,[25,26] this approach has potential drawbacks. *BTK* is a tightly regulated gene with evidence showing that both under- and overexpression can reduce protein efficacy.[27] In addition, the semi-random integration from a viral vector-mediated gene transfer may affect the gene dosage and the regulation of the transgenic BTK depending on the insertion locus. Finally, there remains the risk of insertional oncogenesis despite significant improvements with current generation viral vectors. As an alternative to integrating viral vectors, targeted endonucleases such as Cas9 can be used to integrate a corrective, promoter-less copy of *BTK* at its endogenous locus in the genome and presents an attractive way to sidestep the potential problems associated with viral vectors. Site-specific integration should retain most of the endogenous control of the *BTK* gene, leading to a regulated expression near wild-type levels. Furthermore, using a DSB to guide integration with a highly specific endonuclease should nearly eliminate all off-target integration events and drastically reduce the risk of insertional oncogenesis.

While some genes can be repaired by replacing only specific bases or even small regions of the gene, neither of these approaches are feasible for XLA, as pathogenic mutations span the entire locus without any noteworthy hotspots. Instead, a universal strategy that addresses almost all known disease-causing mutations is to integrate a nearly full-length BTK cDNA donor at the start of the endogenous *BTK* locus. When integrated correctly, this donor supersedes the patient's mutated copy of the gene and leads to an expression of only the corrected transgene. Here, we compare three methods of targeted insertion—HDR, HITI, and PITCh—to achieve integration of a corrective cassette into intron 1 of *BTK* in cell lines and primary human peripheral blood stem cells (PBSCs).

RESULTS

HDR, HITI, and PITCh plasmid donors achieve targeted integration and expression in K562 cells

Corrective BTK cDNA cassettes were designed to integrate at intron 1 because intronic regions are permissive to imperfect junctions that are expected to result from HITI- and PITCh-mediated integrations (Figures 1A and S1). A single-guide RNA (sgRNA) with high specificity to intron 1 of *BTK* was delivered along with Cas9 either from an expression plasmid, as *in vitro*-transcribed mRNA, or was precomplexed to Cas9 protein as a ribonucleoprotein (RNP) to introduce a targeted DSB (Figure 1C). The resultant DSB can then be resolved via various integration pathways (Figure 1B). HDR and PITCh (MMEJ) require end resection to expose homologous regions between the template and the target site and are limited to the S/G_2 or S phases of the cell cycle, respectively.[20] In contrast, HITI (NHEJ) does not require end resection and can occur during any stage of the cell cycle.[28]

Plasmid donor repair templates were assembled to assess the potential for each method of DNA integration to incorporate a therapeutic gene cassette in cell lines (Figures 1D and S6). HDR donors contained the corrective BTK cassette flanked by 500 bp homology arms that parallel the sequences immediately adjacent to the Cas9 cut site. The cassette itself included the BTK cDNA sequence, three C-terminal hemagglutinin (HA) tags to facilitate the detection of the transgene in wild-type cells, a stop codon, and the BTK 3′ untranslated region (UTR) (Figure S1A). Finally, to maintain splicing elements following donor integration, the 3′ region of intron 1 was included preceding the cDNA sequence. This sequence is immediately adjacent to the 5′ homology arm. In the HDR donor ("HDR"), the retention of this intronic sequence also preserved a functional Cas9 binding site, which could be re-cleaved and lead to additional integration events through pathways besides HDR as well as result in unwanted indels following seamless HDR-mediated donor integration. Therefore, a second variant of the HDR donor ("HDR ΔPAM") was generated with a 2 bp modification to the protospacer adjacent motif (PAM) site from NGG to NAA that abrogates Cas9-mediated cleavage of the donor after successful integration.

As HITI-mediated integration requires linearized donor cassettes, donor templates were designed to contain Cas9 binding sites flanking the cDNA sequence to allow for plasmid linearization. CRISPR-Cas9 sites were included at either one or both ends of the donor template in an orientation opposite that found in the target genomic DNA. This reversal of Cas9 binding sites within the donor prevents restoration of sgRNA binding sites upon gene integration in order to eliminate unwanted re-cleavage events (Figure S1D). However, if donor cassettes

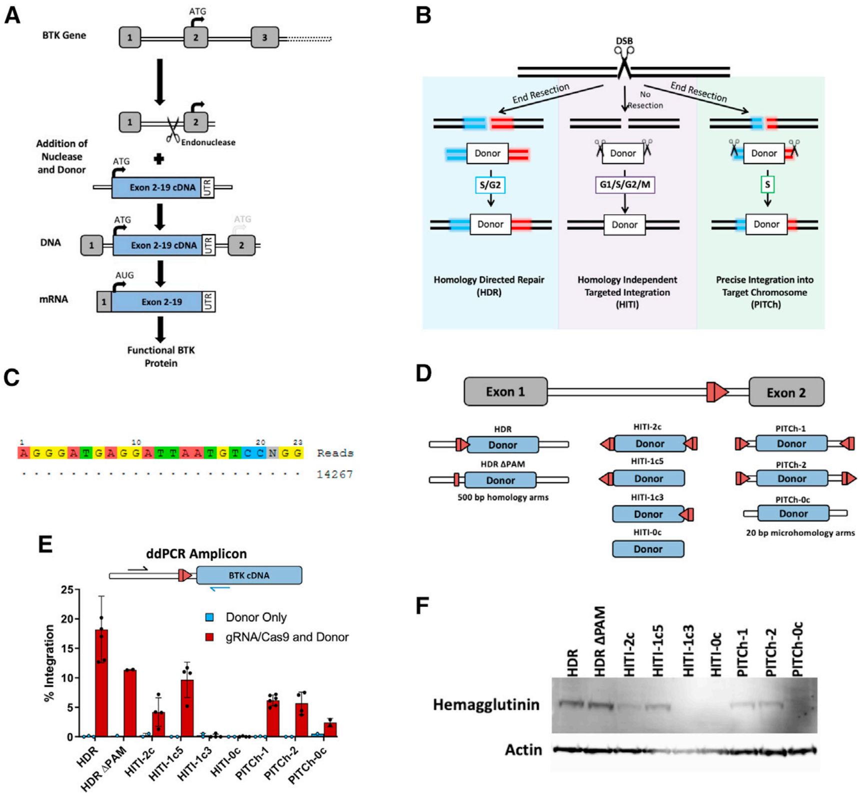

Figure 1. Editing schema and methods of targeted DNA integration

(A) Diagram of proposed editing of the Bruton's tyrosine kinase (*BTK*) gene. Boxes represent exons, while double lines are introns. Cas9/single-guide RNA (sgRNA) targeted to *BTK* intron 1 creates a double-stranded break (DSB) at the 3′ end of the intron. Simultaneous addition of an exogenous donor allows for one of the cell's natural DSB repair pathways to be harnessed for targeted integration of the construct into the open-cut site. The integrated product will lead to RNA transcribed from the corrected *BTK* locus and production of functional protein. (B) DSBs can be repaired through multiple pathways, at least three of which can be harnessed for targeted integration of exogenous DNA. When end resection occurs, homology-directed repair (HDR) or PITCh can use homologous DNA sequences of varying lengths to guide integration into the DSB. These pathways occur predominantly in the S/G_2 and S phases of the cell cycle, respectively. HDR requires longer tracks of homology, while PITCh has short microhomology regions flanked by regions that will bind to the introduced ribonucleoprotein (RNP) and be cut. If no end resection occurs, homology-independent targeted integration (HITI) can be harnessed for integration. HITI donors have no homology to the cut site; however, they are flanked by 1 or 2 cut sites that match the RNP introduced, followed by end capture into the genomic locus. HITI can occur throughout all phases of the cell cycle. (C) Off-target analysis of the sgRNA targeting intron 1 of *BTK* as determined by genome-wide, unbiased identification of DSBs enabled by sequencing (GUIDE-seq). (D) Schematics of the 9 donor variants evaluated. The red arrows together represent sgRNA/Cas9 binding sites with the rectangle representing the 17 bp of the protospacer on one side of the cut site, while the triangle represents the remaining 3 bp of protospacer and the protospacer adjacent motif (PAM). Two HDR donor variants were evaluated that differed only in the presence or absence of an intact PAM sequence. Four HITI donors were designed with sgRNA/Cas9 binding sites on both, either, or neither sides of the corrective donor template. Three PITCh donors were evaluated: two with sgRNA/Cas9 binding sites in differing orientations and one that lacked sgRNA sites entirely. In addition to the donor sequence, each donor contains 3 C-terminal hemagglutinin epitope tags to allow for the identification of transgenic protein products. (E) Diagram of the droplet digital PCR (ddPCR) amplicon used to measure integration rates of each donor template in K562 erythroleukemia cell lines. The reverse primer bound the codon-optimized sequence of the donor template, while the forward primer bound *BTK* intron 1 outside of the 5′ homology arm sequence found in any of the donors. Integration results from each condition are displayed when either the donor plasmid was electroporated into K562 cells alone (blue bars) or in conjunction with the sgRNA/Cas9 expression plasmid (red bars). Error bars represent standard deviation form the mean. (F) Representative immunoblot analysis of cells treated with both sgRNA/Cas9 and the respective donor template listed above. Lysates were probed for the hemagglutinin epitope tags and for an actin loading control.

are end-captured in reverse orientation, re-cleavage can continue until either the transgenic donor integrates in the desired orientation or indels form at the cut site. Although reversal of the Cas9 binding site orientation favors correct integration events, proper integration of this cassette will nevertheless result in a genomic "scar" of either 17 bp (the protospacer side of the cut site) or 6 bp (the PAM side of the cut site), making this strategy most suitable for gene editing approaches at intronic sequences.[18] BTK-specific HITI donors included a cut site on both ends of the corrective cassette ("2c"), one cut site on either the 5′ or 3′ end of the cassette ("1c5" or "1c3"), or no cut sites flanking the cassette ("0c").

PITCh donors contained the corrective cDNA sequence surrounded by microhomology regions of 20 bp that mirror the genomic DNA sequence surrounding the sgRNA cut site, all of which were flanked by two (or no) sgRNA cut sites. The small regions of homology guide the MMEJ repair process, although this donor may also integrate via NHEJ through end-trapping of the donor. Three PITCh donors were assembled: the first ("PITCh-1") had Cas9 binding sites positioned to minimize the length of residual DNA in the donor template after cleavage, the second ("PITCh-2") had both Cas9 binding sites positioned in the same orientation, and finally, a third template ("PITCh-0c") lacked cut sites entirely.

Each donor plasmid was delivered to K562 human erythroleukemia cells via electroporation in conjunction with a Cas9 expression plasmid (pX330) encoding a sgRNA specific to *BTK* intron 1. Four days post-electroporation, gene integration rates of each donor were quantified using droplet digital PCR (ddPCR). The HDR donor achieved the highest integration rates of 18.2% ± 5.6%, while 11.4% ± 0.1% integration was observed using the HDR ΔPAM donor (Figure 1E). The HITI-2c donor resulted in 4.2% ± 2.4% integration

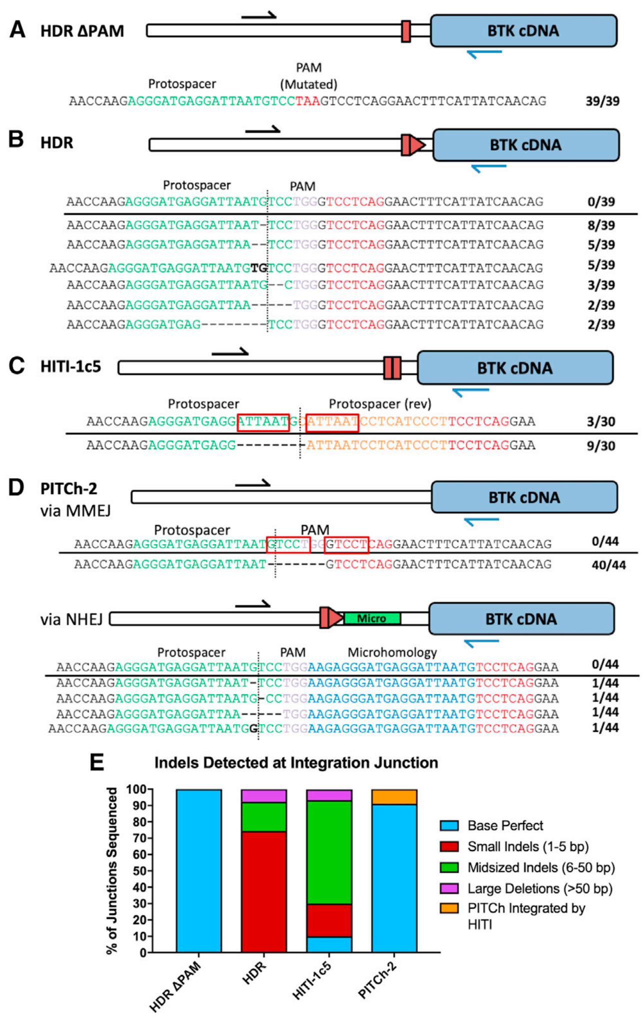

Figure 2. Characterization of donor integration events in BTK deficient K562 cells

BTK-deficient K562 cells were electroporated with the pX330 sgRNA/Cas9 expression plasmid and the corrective donor plasmid. The same primers used for ddPCR described in Figure 1D were used to characterize integration junctions using HDR ΔPAM, HDR, HITI-2c, and PITCh-2 donors. PCR products from each of the conditions were TOPO TA-cloned, and the resulting plasmids were Sanger-sequenced to identify the integration junctions produced by each DNA repair pathway. Sequencing results are shown in (A)–(D). The protospacer from each edit is highlighted in green, while the PAM sequence is in pink if it is intact or in red for the PAM-mutated donor. The sequence "TCCTCAG" is highlighted in red as the putative BTK intron 1 branchpoint. The cut site in the genomic DNA is represented by a vertical dotted line. In the HITI-1c5 donor, integration of the donor template protospacer was detected and is represented here in orange. The PITCh-2 microhomology arm is shown in blue. Inserted bases are shown as bolded text, while deleted bases are dashes. Finally, red boxes were used to illustrate micro-homologous DNA sequences flanking the cut site. (E) Summary of Sanger sequencing data.

compared to 9.7% ± 3.0% using the HITI-1c5 donor. The HITI-1c3 donor had no detectable integration, although it would have produced a much larger amplicon outside the optimal range for detection via ddPCR assay. Likewise, the HITI donor without any cut sites had no detectable integration. Both PITCh-1 and PITCh-2 integrated at comparable rates, 6.2% ± 0.9% and 5.7% ± 1.9% respectively. The PITCh-0c donor achieved integration in 2.4% of cells. An immunoblot analysis of the gene-modified cells post-electroporation demonstrated that the amount of transgenic protein closely followed the pattern of integration seen at the DNA level when probing for the C-terminal HA tags (Figure 1F). These data support the potential use for all three pathways to achieve targeted integration and expression of transgenic constructs in cell lines.

Targeted integration via HDR produces fewer indels at the integration junction compared with HITI or PITCh

To characterize the fidelity of the integration junctions using each of the donors, PCR products produced by primers that amplified on-target cDNA integration events in proper orientation were analyzed by Sanger sequencing (n = 30–44). The HDR ΔPAM integration events all displayed seamless integration junctions, whereas the HDR donor, which contains an intact PAM sequence, yielded no perfect clones (Figures 2A and 2B). Instead, these sequences predominantly featured small indels of 1–5 bp. The HITI-1c5 donor had 10% of sequences with base perfect junctions (Figure 2C). The most commonly found sequence was a single 8 bp deletion in 30% of samples. There is an identical 6 bp sequence, ATTAAT, present on either side of the cut site, and the deleted region corresponds to a deletion of one of those repeats and the intervening bases by a recombination event. The PITCh-2 sequences demonstrated integration via both NHEJ and MMEJ (Figure 2D). NHEJ-mediated integration of the PITCh-2 donor occurred in 9.1% of clones due to the presence of flanking cut sites in the donor. In these cases, the whole construct and the flanking microhomology arms are end-captured, leaving residual bases from two copies of each microhomology arm being integrated. Another 90.9% of the clones had sequences that integrated via MMEJ-mediated integration that resulted in the predicted junction sequence. A summary of indels detected at the integration junction for each of the donors is shown in Figure 2E.

To describe the integration patterns of the different donors, In/Out PCR was performed using primer pairs designed to detect integration and the orientation of the cDNA cassette or plasmid backbone at the 5′ and 3′ junctions (Figure S2A). Each primer pair shared one primer outside of the donor constructs oriented toward the target site, which are represented in black or gray. The first set of primers (black/blue) were identical to those used for ddPCR, and the resulting products demonstrated integration in the correct orientation at the 5′ junction for each of the donors except HITI-1c3, HITI-0c, and PITCh-0c, all of which had no detectable integration by ddPCR (Figure S2B). A primer pair was also designed to amplify only events where the corrective cassette was integrated in reverse orientation (black/red). There were detectable products from cells treated with HITI-2c, HITI-1c5, PITCh-1, and PITCh-2 donors. All four of these donors contain a cut site immediately after the 3′ end of the integration cassette, which would allow for reverse integration of the cassette upon cleavage of the plasmid donor. The final two primer sets

detected integration of the 5′ or 3′ ends of the TOPO 2.1 plasmid backbone at the target site (black/green and black/yellow, respectively). Several of the donors had detectable integrations of the plasmid backbone, and those that yielded the least backbone integration were those without an intact cut site in the donor: HDR ΔPAM, HITI-0C, and PITCh-0c. Of these three donors, only the HDR ΔPAM donor had substantial on-target integration. Similar combinations of primers were used to characterize integration events at the 3′ junction (Figure S2C). The blue/gray primer pair detected reverse integration of the cDNA using donors that contained an intact cut site at the 5′ end of the cDNA donor (HDR, HITI-2c, HITI-1c5, PITCh-1, and PITCh-2). Proper forward integration of the cDNA cassette (red/gray) was detected at the 3′ junction for all except the HITI-0c and PITCh-0 donors. Integration of the plasmid backbone in either direction (green/gray or yellow/gray) was also detected in many samples, notably those with cut sites flanking the cDNA donor. Finally, different combinations of the red and blue primers were used to detect integration of cDNA concatemers (Figure S2D). These events were detected in the HITI-2c, PITCh-1, and PITCh-2 samples, likely because these donors are flanked by sgRNA cut sites on both ends, allowing the cDNA donor to be cut out of the plasmid backbone and integrate in multiples at the *BTK* locus. There was also some concatemerized cDNA product detected using the HDR donor, likely resulting from sequential integration events due to an intact PAM/sgRNA cut site.

Synchronization of K562 cells into G_1 of the cell cycle reduces rates of HDR- and PITCh-based integration while increasing HITI-mediated integration

While K562 cells serve as a valuable tool for basic donor evaluation, they do not fully recapitulate primary human HSCs. HSCs are quiescent and are primarily in G_0, whereas K562s are rapidly cycling and have a much higher prevalence of cells in S/G_2. To evaluate whether these donors may be effective in HSCs, K562 cells were reversibly synchronized to the G_1 phase of the cell cycle using hydroxyurea, an inhibitor of ribonucleotide reductase most active during the S phase. Cells were first treated with hydroxyurea for 24 h and analyzed by flow cytometry to identify changes in the cell cycle (Figure 3A). Treatment with hydroxyurea resulted in a reduction of cells in the G_2/M phases of the cell cycle (Figures 3B and S3). Both hydroxyurea-treated and untreated cells were then electroporated with gRNA/Cas9 expression plasmids and one of the corrective BTK donors (HDR, HDR ΔPAM, HITI-1c5, or PITCh-2). Post-electroporation, cells were cultured in hydroxyurea for 3 days before being transferred to fresh medium lacking hydroxyurea for recovery and outgrowth. Integration rates in each population were measured via ddPCR.

Untreated K562 cells showed an integration pattern similar to previous experiments: the HDR ΔPAM, HDR, and HITI-1c5 donors yielded 11.4% ± 5.4%, 11.4% ± 4.4%, and 7.8% ± 1.9% integration, respectively, while the PITCh-2 donor produced only 3.0% ± 0.0% integration. K562 cells treated with hydroxyurea demonstrated a markedly different pattern of integration despite similar rates of allelic disruption and only modest decreases in viability and fold expansion (Figures 3C–3F). The frequency of integration of the HDR ΔPAM donor decreased to 1.1% ± 1.3%, and the PITCh-2 donor produced 0.7% ± 0.4% integration. Integration rates with the HDR donor dropped less precipitously to 4.9% ± 3.5%, while the HITI-1c5 donor increased substantially to 14.7% ± 5.5% integration. These represent fold changes of 0.43, 0.09, 1.75, and 0.235 for HDR, HDR ΔPAM, HITI-1c5, and PITCh-2, respectively, following hydroxyurea treatment. Expression of the integrated gene cassette was also assessed via immunoblot for C-terminal HA tags and was found to be representative of integration rates as measured by ddPCR (Figure 3G). In/Out PCR was then performed on the hydroxyurea-treated samples, and the resulting products were TOPO TA-cloned and analyzed by Sanger sequencing (n = 45–48) (Figure 3H). Similar to cells not treated with hydroxyurea, HDR ΔPAM integration events once again displayed the highest rate of base perfect sequences (62.5%), although many more small indels were also identified at the integration junction (37.5%). The HDR donor resulted in integration events that were still dominated by small indels at the junction (82.6%) with a small amount of >6 bp indels as well (17.4%). Hydroxyurea-treated cells electroporated with the HITI-2c donor produced a small fraction of base perfect integration events (8.9%), which includes a residual genomic scar by design. NHEJ-mediated PITCh-2 donor integrations were increased compared with unsynchronized, cycling cells.

Hydroxyurea cell-cycle synchronization of peripheral blood stem cells skews DNA repair toward HITI-based integration

To evaluate the effects of hydroxyurea-mediated cell-cycle synchronization on primary peripheral blood stem cells, CRISPR RNP complexes and donor constructs were delivered to PBSC cultured in 0.03 mg/mL hydroxyurea for 24 h pre- and post-electroporation (Figures 4A, 4B, and S4). Similar to K562 cell line experiments above, there were modest decreases in the viability and fold expansion of PBSCs with hydroxyurea as quantified by trypan blue exclusion 1 day after electroporation (Figures 4C and 4D). The addition of hydroxyurea did not appear to affect rates of gene disruption when PBSCs were electroporated with CRISPR sgRNA and Cas9 mRNA (Figure 4E). Given the toxicity of plasmids in primary cells, the best-performing donor for each of the three pathways (HDR ΔPAM, HITI-1c5, and PITCh-2) was packaged as adeno-associated virus (AAV) serotype 6 and was used for the evaluation of site-specific integration in PBSCs. Similar to K562 cell lines, rates of HDR-mediated integration decreased from 17.5% ± 1.9% down to 2.9% ± 0.4%, while HITI-mediated integration increased from 5.4% ± 2.0%–11.7% ± 2.0% with G1 synchronization using hydroxyurea. Rates of gene integration were similar with or without hydroxyurea using the PITCh-2 donor (Figure 4F).

All three repair pathways yield similar integration rates in primitive human HSC populations despite HDR donors achieving the highest integration in bulk-edited human PBSCs

As primitive, engrafting HSCs are thought to be more quiescent and less likely to be actively undergoing cell cycle, the three different DNA repair pathways were evaluated in immunophenotypic stem cell

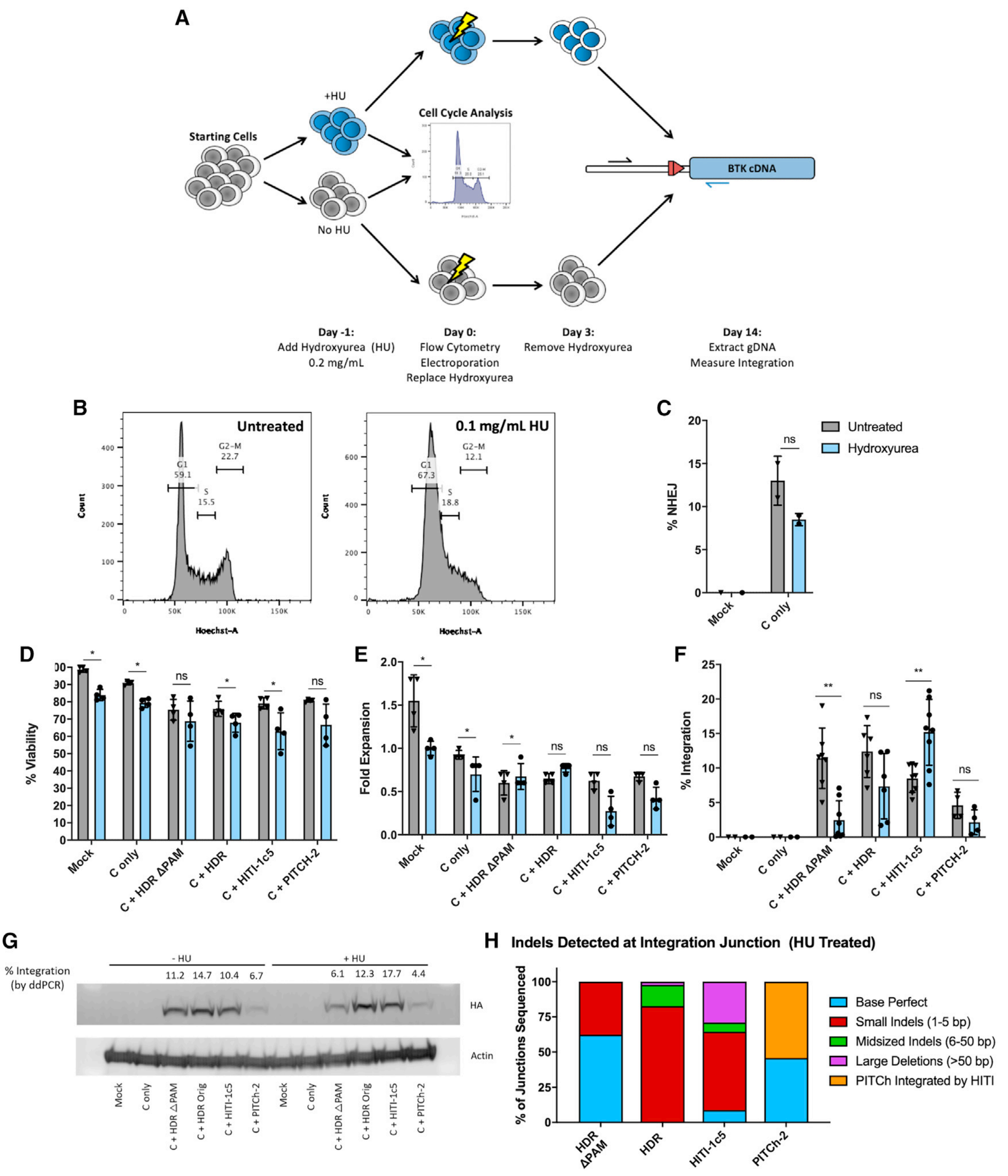

Figure 3. Synchronization of K562 cells in G_1 increases corrective donor integration via HITI while decreasing integration from HDR and PITCh

(A) Schematic of hydroxyurea (HU) synchronization. Cells were cultured with HU for 1 day before electroporation. Immediately preceding electroporation, cells underwent flow cytometric analysis for differences in cell cycle using a Hoechst stain. Following electroporation, cells were cultured for 3 days in media with or without HU, after which HU was removed. Cells were expanded in fresh media for 11 days before analysis for targeted integration via ddPCR. (B) Representative flow cytometry plots for untreated and HU-treated K562 cell populations on day 0. (C) Allelic disruption rates of K562 cells with or without HU culture. (D and E) Viability and fold expansion of cells 1-day post-electroporation were measured by trypan blue exclusion. (F) Targeted integration rates of corrective donors at the *BTK* locus in K562 cells. Gray bars represent cells grown without HU, while blue bars represent cells treated with HU (n = 4–8, 2–4 independent experiments). (G) Representative immunoblot from one of the experiments in Figure 4F detecting hemagglutinin tags (included at the 3′ end of each donor), which serves as a surrogate for expression of the integrated transgene. (H) In/Out PCR products from integration of HDR ΔPAM, HDR, HITI-1c5, and PITCh-2 donors into HU-treated K562 cells were TOPO TA-cloned, and the resulting plasmids were Sanger-sequenced to characterize integration junctions produced by each DNA donor. Data are presented as mean ± SD. Data in (C)–(F) were analyzed by Mann-Whitney test. *$p \leq 0.05$, **$p \leq 0.01$, ns, not significant.

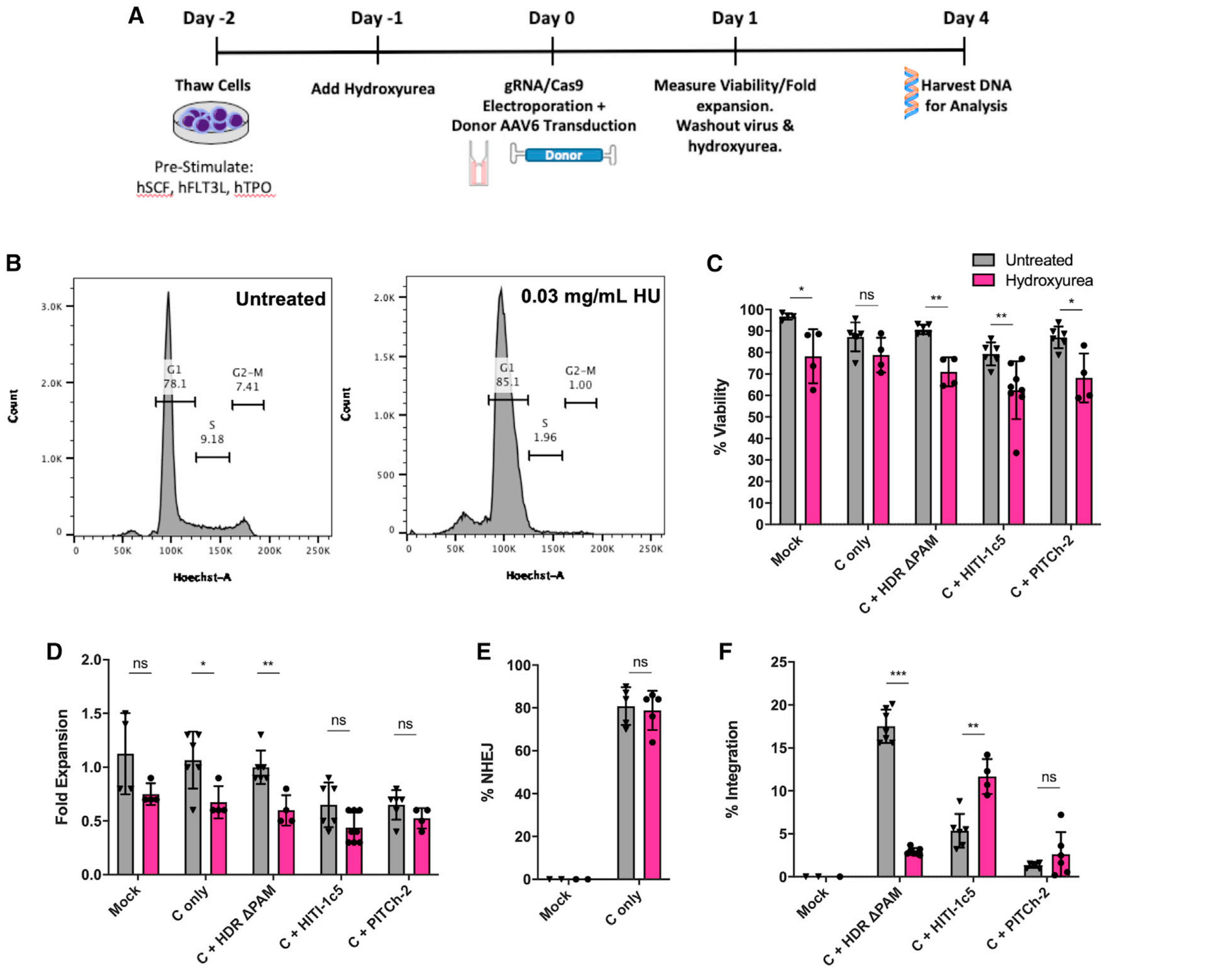

Figure 4. Synchronization of PBSCs in G_1 results in increased corrective donor integration via HITI while decreasing integration via the HDR pathway

(A) Schematic of HU synchronization for peripheral blood stem cells (PBSCs). While in pre-stimulation media 1 day after thaw, 0.03 mg/mL HU was added to the culture. HU was maintained in the media until 1 day after gene modification, when both the AAV and HU were washed out. (B) Representative flow cytometry plots of PBSCs cultured with and without HU. (C and D) Viability (C) and fold expansion (D) of cells 1-day post-electroporation were measured by trypan blue exclusion. (E and F) Allelic disruption rates are shown in (E), while cDNA integration rates as measured by ddPCR are depicted in (F). Data are presented as mean ± SD. Data in (C)–(F) were analyzed by Mann-Whitney test. *$p \leq 0.05$, **$p \leq 0.01$, ***$p \leq 0.001$, ns, not significant.

populations. Mobilized PBSCs from healthy donors were electroporated with gRNA/Cas9 as RNPs following 2 days of pre-stimulation in serum-free media with cytokines. Immediately following electroporation, cells were transduced with one of the three AAV6 donors for 24 h. One week post-electroporation, cells were harvested for genomic DNA and assessed for site-specific integration efficiency (Figure 5A). Three forms of gRNA/Cas9 were assessed in PBSC: chemically synthesized gRNA with chemical modifications (2′-O-methyl analogs and 3′ phosphorothioate internucleotide linkage modifications on the first three 5′ and 3′ terminal RNA residues) with Cas9 mRNA, *in vitro*-transcribed (IVT) gRNA with Cas9 mRNA, and chemically modified gRNA pre-complexed to Cas9 as RNPs (Figure 5B). Although RNPs typically result in higher rates of gene disruption in primary cells, sgRNA targeting this particular sequence in the *BTK* gene consistently achieved the highest rates of allelic disruption when used in conjunction with Cas9 mRNA, regardless of the PBSC source. In addition, different donor multiplicities of infection (MOIs) were tested to maximize gene editing while minimizing toxicity of the donor template (Figure S5). While the HDR donor showed a very clear dose response to increasing MOI, HITI and PITCh donors consistently yielded lower rates of integration. At MOIs above 1×10^5, the fold expansion of cells 24 h after gene editing dropped precipitously despite high viability rates. In addition, in bulk PBSCs, the HDR donor integrated at 21.9% ± 3.6% of cells treated with modified gRNA and Cas9 mRNA. However, the HITI and PITCh donors both demonstrated their highest levels of editing with modified gRNA and Cas9 protein (1.7% ± 0.7% and 6.9% ± 3.5%). These conditions also produced 7.6% ± 2.1% editing

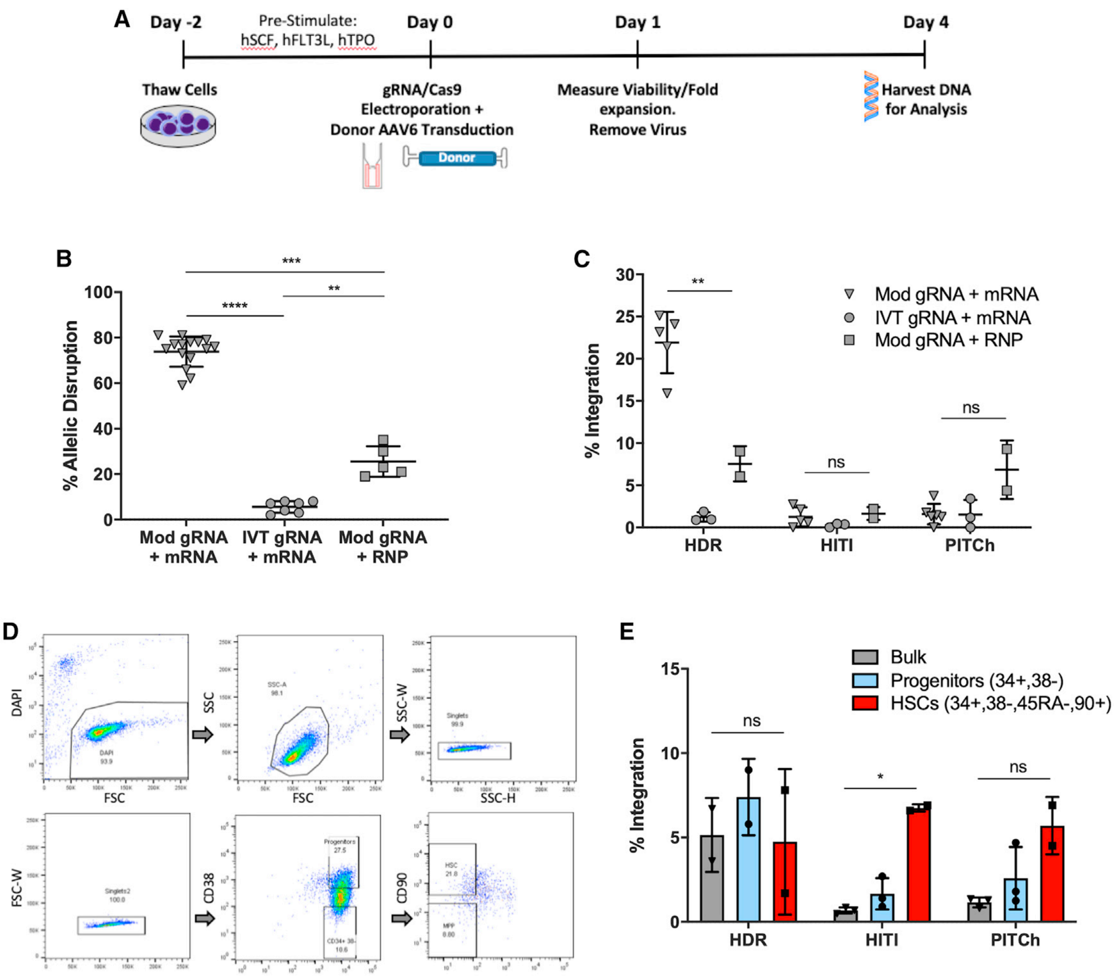

Figure 5. Targeted integration rates in bulk and population-sorted primary human mobilized peripheral blood stem cells

(A) Experimental timeline for gene editing of human mobilized PBSCs. (B) The percetange of allelic disruption as measured by Sanger sequencing (using inference of CRISPR edits [ICE]) in PBSCs using different combinations of sgRNA and Cas9. sgRNA was produced via *in vitro* transcription (IVT) or was chemically synthesized with 2′-O-methyl analogs and 3′ phosphorothioate internucleotide linkages at each of the three 5′ and 3′ terminal RNA residues (labeled “Mod”). Cas9 was delivered either as a protein pre-complexed with the gRNA (RNP) or as messenger RNA (n = 5–15 from 3 PBSC donor sources). (C) Targeted integration rates of each donor type in bulk CD34+ PBSCs. The three best-performing adeno-associated viral (AAV) donor vectors were tested for integration potential. (D) Representative flow cytometry plots demonstrating the sorting schema to differentiate between hematopoietic stem cells (HSCs: CD34+, CD38–, CD45RA–, CD90+) and progenitors (CD34+, CD38+). (E) Targeted integration rates of each donor type in bulk PBSCs (gray), progenitors (blue), or HSCs (red) as measured by ddPCR (n = 2–3 biological replicates from one of the PBSC donor sources from B and C). Cells were treated with chemically synthesized sgRNA pre-complexed to Cas9 as RNPs and AAV6 repair templates. Data are presented as mean ± SD. Data in (B), (C), and (E) were analyzed by Mann-Whitney test. *$p \leq 0.05$, **$p \leq 0.01$, ***$p \leq 0.001$, ****$p \leq 0.0001$, ns, not significant. For (E), p = 0.08 using the HDR donor and p = 0.2 using the PITCh donor.

with the HDR donor (Figure 5C). Taking these results into consideration, modified gRNA with Cas9 as an RNP with a donor MOI of 1×10^5 was used for further comparisons.

To further understand editing in HSPCs, bulk-edited PBSCs were sorted into different HSC subpopulations. Twenty-four hours following CRISPR-Cas9 RNP electroporation and AAV6 transduction, PBSCs were separated via fluorescence-activated cell sorting into two different populations: HSCs (CD34+/CD38−/CD90+/CD45RA−) and progenitors (CD34+/CD38+) (Figure 5D). After expansion in culture, gDNA was harvested and analyzed for donor integration via ddPCR (Figure 5E). The HDR donor integrated in 5.2% ± 2.2% of bulk PBSCs, 7.4% ± 2.3% of sorted progenitors, and 4.8% ± 4.3% of sorted HSCs. The HITI donor yielded integration rates of 0.7% ± 0.2% in bulk PBSCs, 1.7% ± 0.9% in progenitors, and 6.8% ± 0.2% in HSCs. Finally, the PITCh donor produced 1.2% ± 0.3% integration in bulk PBSCs, 2.6% ± 1.9% in progenitors, and 5.7% ± 1.7% in HSCs. Despite large differences in editing efficiency in bulk PBSCs and sorted progenitors, all three methods led to comparable levels of integration in the immunophenotypic HSCs.

DISCUSSION

The results here offer an unbiased comparison of three different methods of targeted integration at the *BTK* locus in cell lines and primary HSPCs. In this study, nine donor variations allowing different DNA repair pathways (HDR, HITI, and PITCh) were evaluated for integration efficiency and integration junction fidelity. In cell lines, HDR-based methods led to the highest levels of integration, likely due to the frequently cycling nature of these cells. HDR, and particularly the HDR donor with a PAM mutation, led to the fewest undesired integration events detected by PCR. The same donor led to 100% base perfect sequence junctions. HITI donors at the same target site had the second highest rates of integration. The addition of only one cut site on the 5′ end of the donor achieved the highest integration rates. Notably, the addition of a second cut site on the 3′ end of the donor led to a drop in integration by about 50%. The addition of a second cut site divides the donor into two similarly sized fragments (one being the corrective cassette and the other being the plasmid backbone), which compete to integrate into available nuclease-mediated DSBs. In this scenario, each fragment would integrate approximately half of the time, thereby decreasing the rate of desirable integrations.

When examining the fidelity of the 5′ integration junctions, some donor variants led to higher rates of precise integration. Notably, the HDR ΔPAM donor yielded only seamless junctions, while the almost identical HDR donor that lacked a PAM had no sequence-perfect junctions. There are likely two different mechanisms that explain the discrepancy that results from only a 2 bp change in these donors. First, the presence of an intact binding site for the gRNA/Cas9 in the donor plasmid made the donor capable of end-capture integration via NHEJ, similar to the HITI donors, resulting in indels in the junction. The second more common mechanism is that even a perfect integration of the HDR donor restores the Cas9 binding site in the genomic DNA, allowing for the endonuclease to re-cleave an already integrated product. In this case, NHEJ could occur following the desired HDR event to produce indels at the junction. For HITI donors, even a "perfect" integration event with no additional indels will leave a scar by design. As expected, all of the HITI donors produced high levels of aberrant integration events via qualitative PCR screening, and the HITI-2c donor had very few base perfect junctions identified via sequencing. Surprisingly, many of the sequenced junctions had a deletion of a region of microhomology, suggesting that they may have integrated via the MMEJ pathway in some cases rather than the NHEJ pathway alone. PITCh donors consistently resulted in the lowest integration rates, although the majority of integration junctions had base perfect sequences.

HDR donors are ideal for use in rapidly cycling cell lines or in genomic loci that are intolerant of indels. However, in instances where homology arms cannot be practically added, such as large payloads approaching the maximum capacity of a delivery vector, using a HITI donor may be an acceptable replacement. It is important to note that these HITI donors leave DNA scarring in the form of duplicated portions of the protospacer or PAM sequences. Therefore, Table 1 HITI donors can only be used effectively in regions or cases where imperfect junctions can be tolerated, such as in introns or UTRs or with the addition of a transgenic promoter that eliminates donor reliance on endogenous sequences for regulation. In this study, PITCh consistently performed the worst of the three tested methods and is not a recommended method of integration in cell lines.

Synchronizing K562 cells into the G_1 phase of the cell cycle with hydroxyurea significantly altered the dynamics of donor integration, reflecting the different mechanisms by which integration can occur based on the cell type and its characteristics. HDR and PITCh donor integration rates both dropped to nearly 0%, while rates of HITI integration approximately doubled. Notably, rates of integration using the HDR donor, which contains an intact PAM site, only dropped by half compared with unsynchronized cells. This may be due to the ability of this particular donor to integrate via a HITI-like pathway upon cleavage of the donor; this is in contrast to the HDR ΔPAM donor, which lacks an intact cut site and can only integrate via HDR. Hydroxyurea synchronization of PBSCs also yielded similar results, reducing rates of HDR while increasing rates of HITI-mediated gene modification. Together, these experiments support the cell-cycle dependencies of each pathway and are consistent with the initial hypothesis that HITI may be an ideal mechanism for targeted integration for non-replicating cells.

In primary human PBSCs, the optimized donor for each integration method led to predicted integration rates in bulk cells. HDR donors achieved the highest overall integration rates, likely due to the cycling, larger proportion of more differentiated progenitor cells that compose the bulk population. Gene correction in cycling progenitor cells possesses less therapeutic relevance because these cells are less likely to engraft long term and self-renew like true HSCs. While HDR vastly outperformed the two other pathways in bulk cells, all three donors led to similar levels of targeted integration in immunophenotypic HSCs. In fact, the percentage of gene integration was either maintained or significantly higher in sorted CD34+CD38−CD90+CD45RA− HSCs compared with bulk-edited PBSCs across all donor types. The ability of the PITCh donor to achieve similar rates of integration in HSCs was unexpected due to MMEJ's dependence on the S phase of the cell cycle. One possible explanation for this finding is that the PITCh donor can also integrate via HITI due to the presence of flanking cut sites just beyond the microhomology arms.

In all, these findings suggest that HDR-, HITI-, and PITCh-mediated targeted integrations can be effective for editing in primary human HSCs. While levels of bulk editing vary substantially, integration rates are nearly equivalent in immunophenotypic HSCs. Integration into an exon or sequence that requires a seamless junction is likely best approached with an HDR donor. However, integration into non-coding regions may tolerate all three approaches. HITI and PITCh donors do not require the full homology arms present in an HDR donor, which can conserve the sequence length in larger donors that are nearing the size limits for viral packaging. At the BTK locus, HITI donors

Table 1. Droplet Digital PCR Primer and Probe Sequences

ddPCR primer/Probe	Sequence
BTK fwd	5′-AGCAGTTAGTGTGTGTCCAGAAC-3′
BTK rev	5′-ATCTTTTCCACGTCGATGCT-3′
BTK probe	5′-TCGAAGTCGTACTC GTAGTAGCTCAGCTTG-3′
UCE35682 fwd	5′-CTAATGTGTCCCTTTTGAGT-3′
UCE35682 rev	5′-CAAACAGTTTTGATGAAGCT-3′
UCE35682 probe	5’ HEX-TGCCACCCC-ZEN-TGTCTGATTGTGT- 3′ Iowa

Fwd, forward; rev, reverse.

achieved similar or better results than PITCh donors in each experiment, making it the preferable alternative to HDR. However, the ideal length of microhomology arms is also not known for MMEJ-mediated integration and may have required additional optimization for BTK. While there may be some locus dependent variables that impact the efficiency of the different repair pathways, the data presented here support the potential for all three pathways to lead to functional integration in HSCs and to act as potential avenues for clinically relevant gene therapies.

MATERIALS AND METHODS

gRNA design

sgRNAs targeting BTK intron 1 were identified using the design algorithm from CRISPRscan.[29] sgRNAs were assembled and cloned into a gRNA/Cas9 expression plasmid (pX330) (Addgene plasmid #42230).[2] The sgRNA targeted the sequence 5′-AGGGATGAGGATT AATGTCC-3′ at the 3′ end of *BTK* intron 1.

Donor design

HDR donors contained *BTK* cDNAs from exons 2 to 19 that were codon-optimized using the GeneOptimizer online tool (Thermo Fisher Scientific, Waltham, MA, USA). BTK exon 2 has a small portion of an untranslated sequence that was included in each donor but was not codon-optimized, as there are no coding regions in this sequence. The donor template contains the 3′ splice site from the end of *BTK* intron 1, the exon 2 portion of the 5′ UTR, codon-optimized exon 2–19 *BTK* cDNA, three C-terminal HA tags, a stop codon, the BTK 3′ UTR, and a short sequence to improve polyadenylation.[30] HDR donors were flanked by homology arms of 500 bp that match the sequences immediately adjacent to the target site in the genomic DNA. PITCh donors were flanked by 20 bp of a homologous sequence. HITI donors had no homologous flanking regions. HDR ΔPAM had a 2 bp mutation in the PAM sequence (NGG to NAA). For all cell line experiments, the donors were delivered as plasmids.

K562 electroporation and hydroxyurea synchronization

2×10^5 K562 cells per condition were electroporated with 500 ng of sgRNA/Cas9 expression plasmid and 3 ug of the respective donor plasmid using the Lonza 4D Nucleofector X Unit (Cat: AAF-1002X, Lonza, Basel, Switzerland) in 20 uL of Lonza SF buffer (Cat: V4XC-2032, Lonza, Basel, Switzerland). After electroporation, cells were plated in 500 uL of R10 media (RPMI 1640; Cat: 15-040-CV, Corning, Corning, NY, USA) supplemented with 1× penicillin/streptomycin/glutamine (Cat: 10378016, ThermoFisher Scientific, Waltham, MA, USA) and 10% fetal bovine serum (Omega Scientific, Tarzana, CA, USA). For cell-cycle synchronization, K562 cells were pre-treated with 100 ug/mL of hydroxyurea for 1 day before nucleofection (Cat: H8627; Millipore Sigma, Burlington, MA, USA). Cell-cycle analysis was performed using Hoechst dye (Cat: 565877; BD Biosciences, San Diego, CA, USA) following manufacturer protocol. Three days after electroporation, treated cells were washed and re-suspended in R10 without hydroxyurea. Genomic DNA was harvested from the cells using the Invitrogen PureLink Genomic DNA Mini Kit (Cat: K182002; ThermoFisher Scientific, Waltham, MA, USA). Protein lysates from 2×10^6 cells per condition were harvested 1 to 3 weeks post-nucleofection using Denaturing Cell Extraction Buffer (Cat: FNN0091; ThermoFisher Scientific, Waltham, MA, USA) + 1× Halt Protease Inhibitor (Cat: 87786; ThermoFisher Scientific, Waltham, MA, USA). Lysate concentrations were quantified via bicinchoninic acid (BCA) assay (Cat: 23225; ThermoFisher Scientific, Waltham, MA, USA).

Quantifying targeted integration

Integration rates were measured by ddPCR. *BTK* specific primers and probes are listed in Table 1. A reference primer/probe set recognized the UC335682 region of the X chromosome. Integration rates were quantified using the QX200 BioRad Droplet Reader (Cat: 186–4003; BioRad, Hercules, CA, USA).

Immunoblotting

Equal amounts of protein lysates per sample were loaded in each well of 4%–12% Bis-Tris gel (Cat: NP0322BOX; Thermo Fisher Scientific, Waltham, MA, USA). A primary antibody for HA (Cat: NBP2-43714; Novus Biologicals, Centennial, CO, USA) was used to detect integrated protein, while beta actin was used as a loading control (Cat: MA5-15739; Thermo Fisher Scientific, Waltham, MA, USA). Secondary antibodies were conjugated with horseradish peroxidase (Cat: 31460; ThermoFisher Scientific, Waltham, MA, USA) for the HA secondary and Alexa Fluor 647 for the actin secondary (Cat: A-21236; ThermoFisher Scientific, Waltham, MA, USA) at dilution factors of 1:750 for HA and 1:1,000 for actin.

Human peripheral blood stem cell electroporations

Human CD34+ granulocyte colony-stimulating factor (G-CSF) mobilized peripheral blood stem and progenitor cells were thawed 2 days prior to electroporation. Cells were pre-stimulated at a density of 5×10^5 cells/mL in X-Vivo 15 medium (Cat: BEBP04-744Q; Lonza, Basel, Switzerland) supplemented with 1× penicillin/streptomycin/glutamine (Cat: 10378016; ThermoFisher Scientific, Waltham, MA, USA), 50 ng/uL recombinant human (rh) stem cell factor (Cat: 300-07; PeproTech, Rocky Hill, NJ, USA), 50 ng/uL rh Flt-3 ligand (Cat: 300-19; PeproTech, Rocky Hill, NJ, USA), and 50 ng/uL rh thrombopoietin (Cat: 300-18; PeproTech, Rocky Hill, NJ, USA). After

2 days of pre-stimulation, cells were electroporated using the BTX system (BTX, Holliston, MA, USA). 2×10^5 cells/condition were electroporated in 100 uL of BTX buffer (255 V, 30 ms, 1 pulse). For RNP samples, 9 μg of chemically modified sgRNA (Synthego, Silicon Valley, CA, USA) were incubated with 200 pmol of Cas9 protein for 20 min at room temperature to make RNP complexes. For Cas9 mRNA electroporations, 5 μg of the chemically modified sgRNA was introduced with 5 μg of SpCas9 mRNA. Following electroporation, samples were transduced with the relevant AAV6 donor vector (Vigene Biosciences, Rockville, MD, USA). One day post-electroporation, cells were resuspended in 500 uL of outgrowth media: IMDM (Cat: 12440053; ThermoFisher Scientific, Waltham, MA, USA), 10% fetal bovine serum, 1× penicillin/streptomycin/glutamine (Cat: 10378016; ThermoFisher Scientific, Waltham, MA, USA), 5 ng/mL of rhIL-3, 10 ng/mL of rhIL-6, and 25 ng/mL of rhSCF (Cat: 200-03, 200-06, and 300-07, respectively; PeproTech, Rocky Hill, NJ, USA). Four days post-electroporation, cells were harvested for analysis. In experiments involving cell-cycle synchronization, PBSCs were cultured in 0.03 mg/mL hydroxyurea for 24 h pre- and post-electroporation.

Sorting human HSPCs into different stem populations

Cells were sorted into two populations: HSC (CD34+, CD38−, CD45RA−, CD90+) and progenitors (CD34+, CD38+) (Cat: 328123 and 304121; Biolegend, San Diego, CA, USA; Cat: 555824 and 555460, BD Biosciences, Franklin Lakes, NJ, USA). Three to ten days post-sort, gDNA was extracted from each condition for analysis.

SUPPLEMENTAL INFORMATION

Supplemental information can be found online at https://doi.org/10.1016/j.omtn.2021.12.014.

ACKNOWLEDGMENTS

The authors thank the UCLA Broad Stem Cell Center Flow Cytometry Core for their assistance with these studies. The authors thank Jessica Scholes, Felicia Codrea, and Jeffrey Calimlim for their expertise and assistance. This work was supported by research grants from the Ruth L. Kirschstein National Research Service Awards AI060567 and GM007185, the Broad Foundation Innovative Pilot Stem Cell Research Project Award, and the NIH NCATS UCLA CTSI grant number UL1TR0001881.

AUTHOR CONTRIBUTIONS

Conceptualization, D.H.G.; methodology, D.H.G., J.S., A.G.K., and I.V.; investigation, D.H.G., J.S., A.G.K., I.V., S.M., E.C.T., J.D.L., and C.Y.K.; resources, C.Y.K.; writing – original draft, D.H.G.; writing – review & editing, D.H.G., J.D.L., and C.Y.K.; visualization, D.H.G.; supervision, C.Y.K.; funding acquisition, D.H.G. and C.Y.K.

DECLARATION OF INTERESTS

The authors declare no competing interests.

REFERENCES

1. Jinek, M., Chylinski, K., Fonfara, I., Hauer, M., Doudna, J.A., and Charpentier, E. (2012). A programmable dual-RNA – guided DNA endonuclease in adaptive bacterial immunity. Science *337*, 816–822.
2. Cong, L., Ran, F.A., Cox, D., Lin, S., Barretto, R., Habib, N., Hsu, P.D., Wu, X., Jiang, W., Marraffini, L.A., and Zhang, F. (2013). Multiplex genome engineering using CRISPR/Cas systems. Science *339*, 819–823.
3. Metzger, L., and Iliakis, G. (1991). Kinetics of DNA double-strand break repair throughout the cell cycle as assayed by pulsed field gel electrophoresis in CHO cells. Int. J. Radiat. Biol. *59*, 1325–1339.
4. Kim, S., Kim, D., Cho, S.W., Kim, J., and Kim, J.-S. (2014). Highly efficient RNA-guided genome editing in human cells via delivery of purified Cas9 ribonucleoproteins. Genome Res. *24*, 1012–1019.
5. Bauer, D.E., Kamran, S.C., Lessard, S., Xu, J., Fujiwara, Y., Lin, C., Shao, Z., Canver, M.C., Smith, E.C., Pinello, L., et al. (2013). An erythroid enhancer of BCL11A subject to genetic variation determines fetal hemoglobin level. Science *342*, 253–257.
6. Bjurström, C.F., Mojadidi, M., Phillips, J., Kuo, C., Lai, S., Lill, G.R., et al. (2017). Reactivating fetal hemoglobin expression in human adult erythroblasts through BCL11A knockdown using targeted endonucleases. Mol. Ther. Nucleic Acids *5*, e351.
7. Cradick, T.J., Fine, E.J., Antico, C.J., and Bao, G. (2013). CRISPR/Cas9 systems targeting β-globin and CCR5 genes have substantial off-target activity. Nucleic Acids Res. *41*, 9584–9592.
8. Hoban, M.D., Cost, G.J., Mendel, M.C., Romero, Z., Kaufman, M.L., Joglekar, A.V., Ho, M., Lumaquin, D., Gray, D., Lill, G.R., et al. (2015). Correction of the sickle-cell disease mutation in human hematopoietic stem/progenitor cells. Blood. https://doi.org/10.1182/blood-2014-12-615948.
9. Kuo, C.Y., Long, J.D., Campo-Fernandez, B., de Oliveira, S., Cooper, A.R., Romero, Z., Hoban, M.D., Joglekar, A.V., Lill, G.R., Kaufman, M.L., et al. (2018). Site-specific gene editing of human hematopoietic stem cells for X-linked hyper-IgM syndrome. Cell Rep. *23*, 2606–2616.
10. De Ravin, S.S., Reik, A., Liu, P.-Q., Li, L., Wu, X., Su, L., Raley, C., Theobald, N., Choi, U., Song, A.H., et al. (2016). Targeted gene addition in human CD34(+) hematopoietic cells for correction of X-linked chronic granulomatous disease. Nat. Biotechnol. *34*, 424–429.
11. Genovese, P., Schiroli, G., Escobar, G., Di Tomaso, T., Firrito, C., Calabria, A., Moi, D., Mazzieri, R., Bonini, C., Holmes, M.C., et al. (2014). Targeted genome editing in human repopulating haematopoietic stem cells. Nature *510*, 235–240.
12. Schiroli, G., Genovese, P., Castiello, M.C., Capo, V., Gregory, P.D., Holmes, M.C., Sitia, G., Villa, A., Lombardo, A., and Naldini, L. (2016). 481. Targeted genome editing in Mouse hematopoietic stem/progenitor cells (HSPC) to model gene correction of SCID-X1. Mol. Ther. *23*, S191.
13. Wang, J., Exline, C.M., DeClercq, J.J., Llewellyn, G.N., Hayward, S.B., Li, P.W.-L., Shivak, D.A., Surosky, R.T., Gregory, P.D., Holmes, M.C., et al. (2015). Homology-driven genome editing in hematopoietic stem and progenitor cells using ZFN mRNA and AAV6 donors. Nat. Biotechnol. *33*, 1256–1263.
14. Branzei, D., and Foiani, M. (2008). Regulation of DNA repair throughout the cell cycle. Nat. Rev. Mol. Cell Biol. *9*, 297–308.
15. Lomova, A., Clark, D.N., Campo-Fernandez, B., Flores-Bjurström, C., Kaufman, M.L., Fitz-Gibbon, S., Wang, X., Miyahira, E.Y., Brown, D., DeWitt, M.A., et al. (2019). Improving gene editing outcomes in human hematopoietic stem and progenitor cells by temporal control of DNA repair. Stem Cells *37* (2), 284–294.
16. Pietras, E.M., Warr, M.R., and Passegué, E. (2011). Cell cycle regulation in hematopoietic stem cells. J. Cell Biol. *195*, 709–720.
17. Shin, J.J., Schröder, M.S., Caiado, F., Wyman, S.K., Bray, N.L., Bordi, M., Dewitt, M.A., Vu, J.T., Kim, W.T., Hockemeyer, D., et al. (2020). Controlled cycling and quiescence enables efficient HDR in engraftment-enriched adult hematopoietic stem and progenitor cells. Cell Rep. https://doi.org/10.1016/j.celrep.2020.108093.
18. Suzuki, K., Tsunekawa, Y., Hernandez-Benitez, R., Wu, J., Zhu, J., Kim, E.J., Hatanaka, F., Yamamoto, M., Araoka, T., Li, Z., et al. (2016). In vivo genome editing via CRISPR/Cas9 mediated homology-independent targeted integration. Nature *540*, 144–149.

19. Nakade, S., Tsubota, T., Sakane, Y., Kume, S., Sakamoto, N., Obara, M., et al. (2014). Microhomology-mediated end-joining-dependent integration of donor DNA in cells and animals using TALENs and CRISPR/Cas9. Nat. Commun. *5*, 5560.

20. Truong, L.N., Li, Y., Shi, L.Z., Hwang, P.Y.-H., He, J., Wang, H., Razavian, N., Berns, M.W., and Wu, X. (2013). Microhomology-mediated end joining and homologous recombination share the initial end resection step to repair DNA double-strand breaks in mammalian cells. Proc. Natl. Acad. Sci. U S A *110*, 7720–7725.

21. Futatani, T., Miyawaki, T., Tsukada, S., Hashimoto, S., Kunikata, T., Arai, S., Kurimoto, M., Niida, Y., Matsuoka, H., Sakiyama, Y., et al. (1998). Deficient expression of Bruton's tyrosine kinase in monocytes from X-linked agammaglobulinemia as evaluated by a flow cytometric analysis and its clinical application to carrier detection. Blood *91*, 595–602.

22. Vetrie, D., Vorechovsky, I., Sideras, P., Holland, J., Davies, A., Flinter, F., Hammarström, L., Kinnon, C., Levinsky, R., Bobrow, M., et al. (1993). The gene involved in X-linked agammaglobulinaemia is a member of the src family of protein-tyrosine kinases. Nature *361*, 226–233.

23. Siegal, F.P., Pernis, B., and Kunkel, H.G. (1971). Lymphocytes in human immunodeficiency states: a study of membrane-associated immunoglobulins. Eur. J. Immunol. *1*, 482–486.

24. Bruton, O.C. (1952). Agammaglobulinemia. Pediatrics *9*, 722–728.

25. Ng, Y.Y., Baert, M.R.M., Pike-Overzet, K., Rodijk, M., Brugman, M.H., Schambach, A., Baum, C., Hendriks, R.W., Van Dongen, J.J., and Staal, F.J. (2010). Correction of B-cell development in Btk-deficient mice using lentiviral vectors with codon-optimized human BTK. Leukemia *24*, 1617–1630.

26. Sather, B.D., Ryu, B.Y., Stirling, B.V., Garibov, M., Kerns, H.M., Humblet-Baron, S., Astrakhan, A., and Rawlings, D.J. (2011). Development of B-lineage predominant lentiviral vectors for use in genetic therapies for B cell disorders. Mol. Ther. *19*, 515–525.

27. Satterthwaite, A.B., Cheroutre, H., Khan, W.N., Sideras, P., and Witte, O.N. (1997). Btk dosage determines sensitivity to B cell antigen receptor cross-linking. Proc. Natl. Acad. Sci. U S A *94*, 13152–13157.

28. Aylon, Y., Liefshitz, B., and Kupiec, M. (2004). The CDK regulates repair of double-strand breaks by homologous recombination during the cell cycle. EMBO J. https://doi.org/10.1038/sj.emboj.7600469.

29. Moreno-Mateos, M.A., Vejnar, C.E., Beaudoin, J., Fernandez, J.P., Mis, E.K., Khokha, M.K., and Giraldez, A.J. (2015). CRISPRscan: designing highly efficient sgRNAs for CRISPR-Cas9 targeting in vivo. Nat. Methods *12*, 982–988.

30. Levitt, N., Briggs, D., Gil, A., and Proudfoot, N.J. (1989). Definition of an efficient synthetic poly(A) site. Genes Dev. *3*, 1019–1025.

OMTN, Volume *27*

Supplemental information

A comparison of DNA repair pathways to achieve a site-specific gene modification of the Bruton's tyrosine kinase gene

David H. Gray, Jasmine Santos, Alexandra Grace Keir, Isaac Villegas, Simon Maddock, Edward C. Trope, Joseph D. Long, and Caroline Y. Kuo

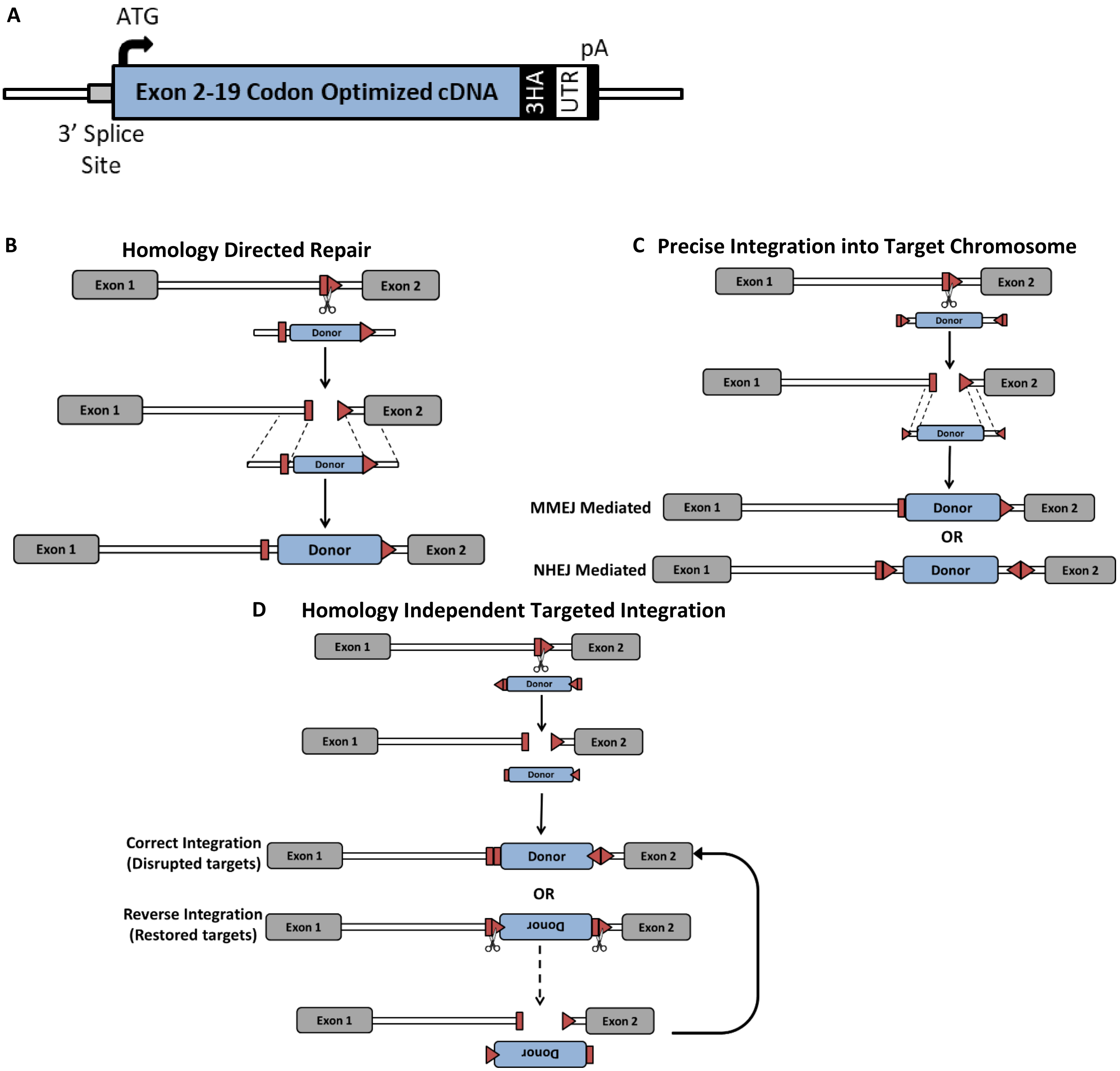

Supplemental Figure 1: Schematics of donor template and targeted integration pathways

A) Detailed diagram of an HDR donor template. Each donor has the full *BTK* coding sequence (exons 2-19) that have been codon optimized. There are 3 C terminal hemagglutinin (3HA) tags immediately after the coding sequence before the stop codon. Following the stop codon is a copy of the *BTK* 3' untranslated region (3UTR) including the poly adenylation site (pA). On the 5' end of the donor sequence is the 3' splice site from *BTK* intron 1 to allow for efficient splicing of the transgenic sequence. **B)** Targeted integration via homology directed repair (HDR). The red rectangle represents the 17 bp of the protospacer that are PAM (protospacer adjacent motif) distal, while the red triangle represents the 3 PAM proximal bp and the PAM. When both pieces are together in the correct orientation (a red arrow), there is an intact target site matching the delivered single guide RNA (represented by scissors). A cDNA donor template flanked by 500 bp of sequence homologous to the region surrounding the double stranded break can be integrated via the HDR pathway. **C)** Integration via precise integration into target chromosome (PITCh). A donor template was

generated flanked by 20 bp of microhomology matching the regions adjacent to the target DSB. Beyond the microhomology regions were two Cas9 binding sites matching the target site in the genomic DNA. As the DSB is generated in the genomic DNA, the target sites in the donor template can also be cleaved. This cleaved donor template can integrate into the genomic DNA DSB via either microhomology mediated end joining (MMEJ) or non-homologous end joining (NHEJ). MMEJ integration is expected to leave a seamless repair while NHEJ integration would produce a duplicate of the microhomology regions and potentially a portion of the donor template's Cas9 binding regions. **D)** Integration via homology independent targeted integration. A donor template with no added homology arms was flanked by one or two Cas9 binding sites that are each the reverse complement of the sequence found in the genomic DNA. Upon delivery of the reagents, both the genomic DNA and the donor template will be cleaved and the resulting cleaved donor can be integrated via NHEJ. Without homology to guide the directionality of the integration, both the in frame and reverse oriented integration events are possible. However, if the donor is integrated in reverse, the Cas9 binding sites will be restored, barring insertions or deletions (indels) at the junction. The integrated donor may then be excised until it orients in the forward orientation or indels form at either junction, preventing re-cleavage.

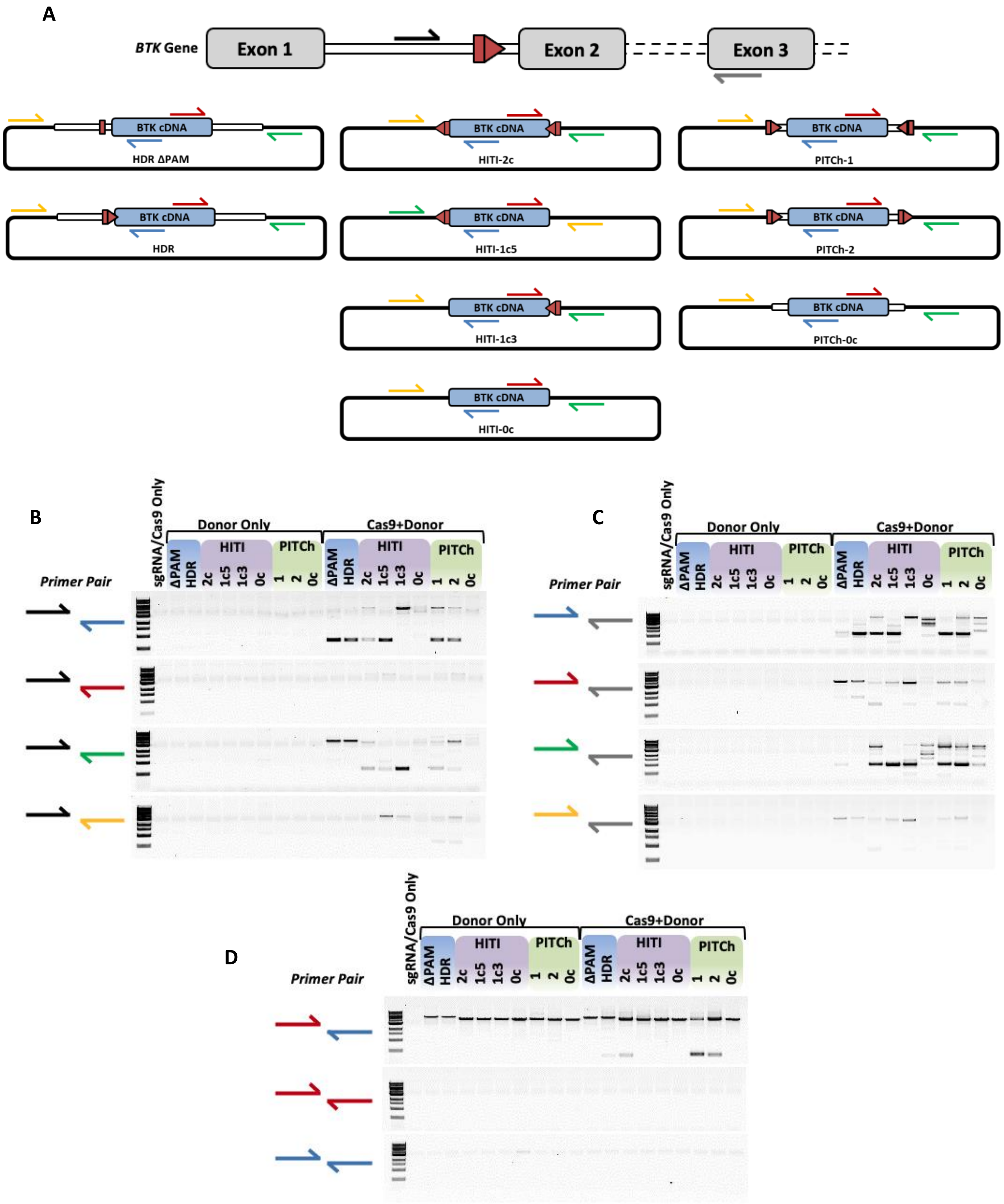
A
BTK Gene
Exon 1
Exon 2
Exon 3
BTK cDNA
HDR ΔPAM
HDR
HITI-2c
HITI-1c5
HITI-1c3
HITI-0c
PITCh-1
PITCh-2
PITCh-0c
B
C
D
Primer Pair
sgRNA/Cas9 Only
Donor Only
Cas9+Donor
ΔPAM
HDR
HITI
2c
1c5
1c3
0c
PITCh
1
2
0c

Supplemental Figure 2: Qualitative evaluation of 5' integration junctions in K562 cells treated with HDR, HITI, or PITCh editing reagents.

A) Schematic showing primers (arrows) used for In/Out PCR of the 5' and 3' integration junctions and their respective binding sites on each donor template. The black primer is specific to the negative strand of *BTK* intron 1 genomic DNA while the gray primer binds to the positive strand in exon 3 just beyond the homology arm to preclude binding of unintegrated donor template. These primers were used as the "Out" primer for each of the In/Out PCRs. The blue primer binds to the to the positive strand of the 5' end of the codon optimized *BTK* cDNA donor sequence, and is the same primer used in droplet digital PCR assays. The red primer binds the negative strand of the 3' end of the codon optimized donor sequence. The green and yellow primers bind to the TOPO 2.1 plasmid backbone on either side of the donor cassette. Due to the flexible directionality of the TOPO TA cloning process used to assemble these donor templates, the HITI 1c5 donor cassette is oriented opposite the other donors in its backbone, meaning the relative positions of the green and yellow primer binding sites are flipped for that donor template. Genomic DNA from K562 cells treated with either sgRNA/Cas9 expression plasmid alone, one of the donor template plasmids alone, or both together was amplified to detect different integration events at the **B)** 5' and **C)** 3' junctions. The different primer pairs detect integration of the cDNA in either forward or reverse orientation or integration of the plasmid backbone in either orientation. **D)** The red and blue primer pairs were also used in different combinations to detect integration of donor concatamers. 10-50 ng of genomic DNA was used in the reactions above with 0.5 uM of each primer and 30-35 PCR cycles based on manufacturer's protocol for Platinum SuperFi II Green PCR Master Mix (ThermoFisher Scientific; Waltham, MA).

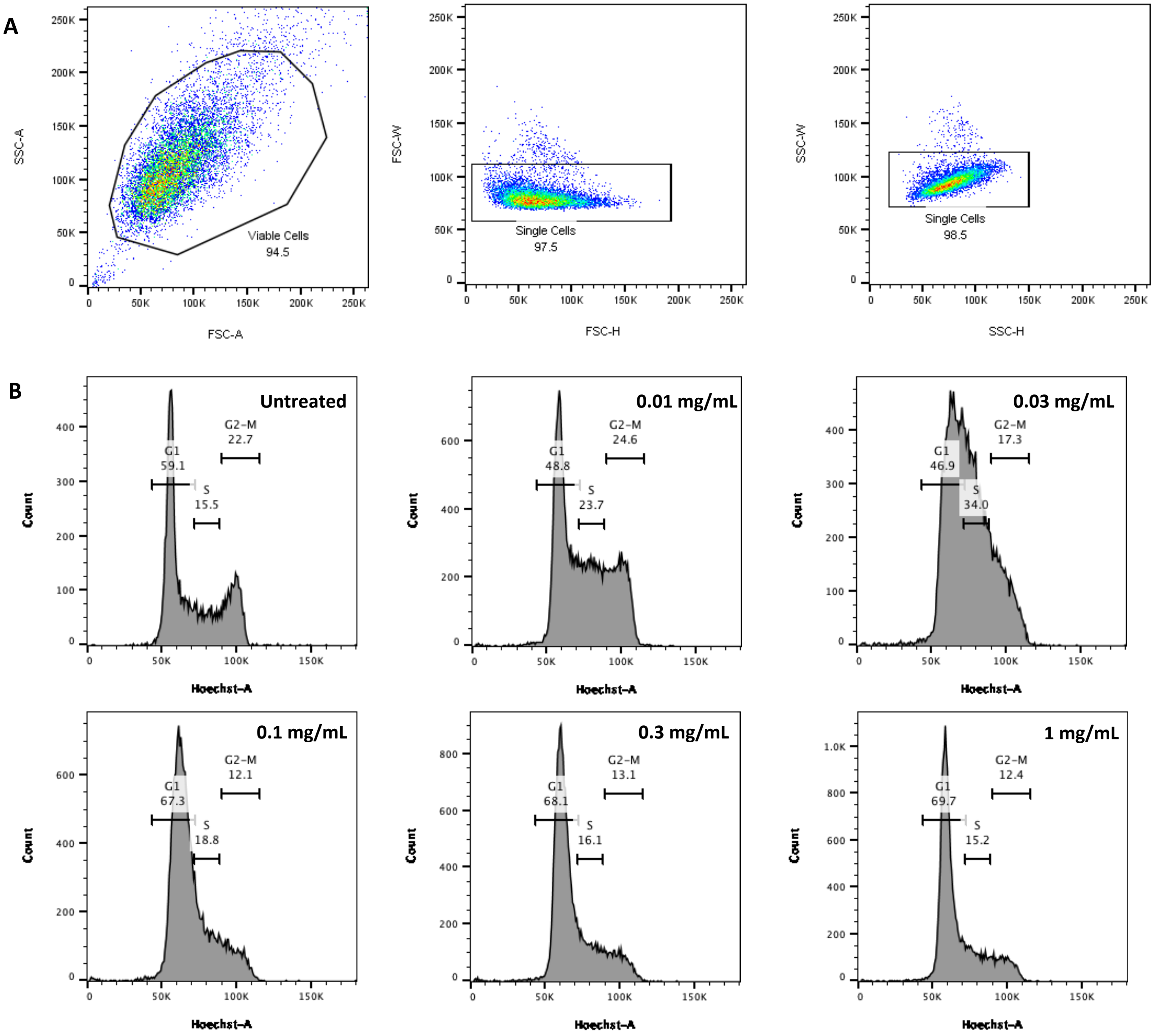

Supplemental Figure 3: Hydroxyurea cell cycle synchronization efficacy in K562 cells

A) Representative gating scheme for viability and single cells during cell cycle analysis. **B)** Hoechst dye analysis of cell cycle performed on K562 cells after incubation with increasing doses of hydroxyurea (HU).

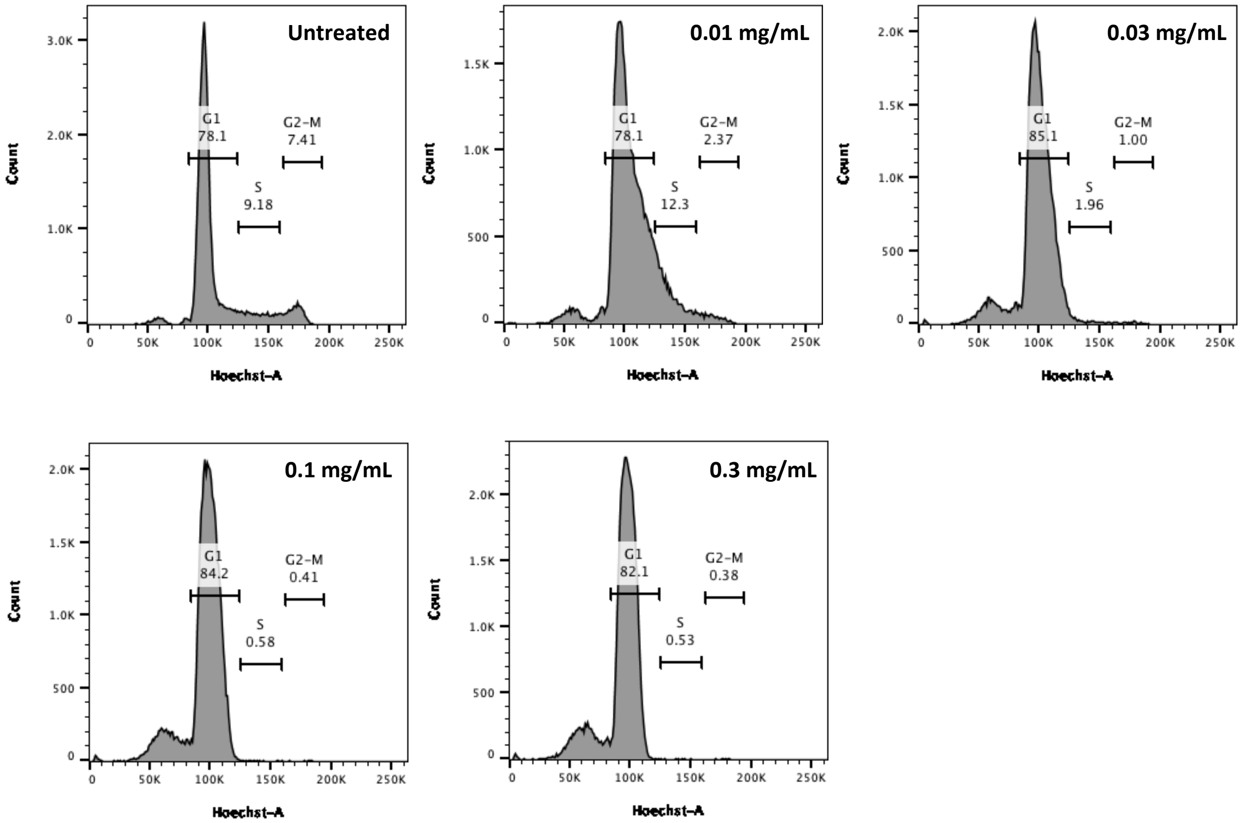

Supplemental Figure 4: Hydroxyurea cell cycle synchronization in PBSC

Hoechst dye analysis of cell cycle performed on PBSC after incubation with increasing doses of hydroxyurea (HU).

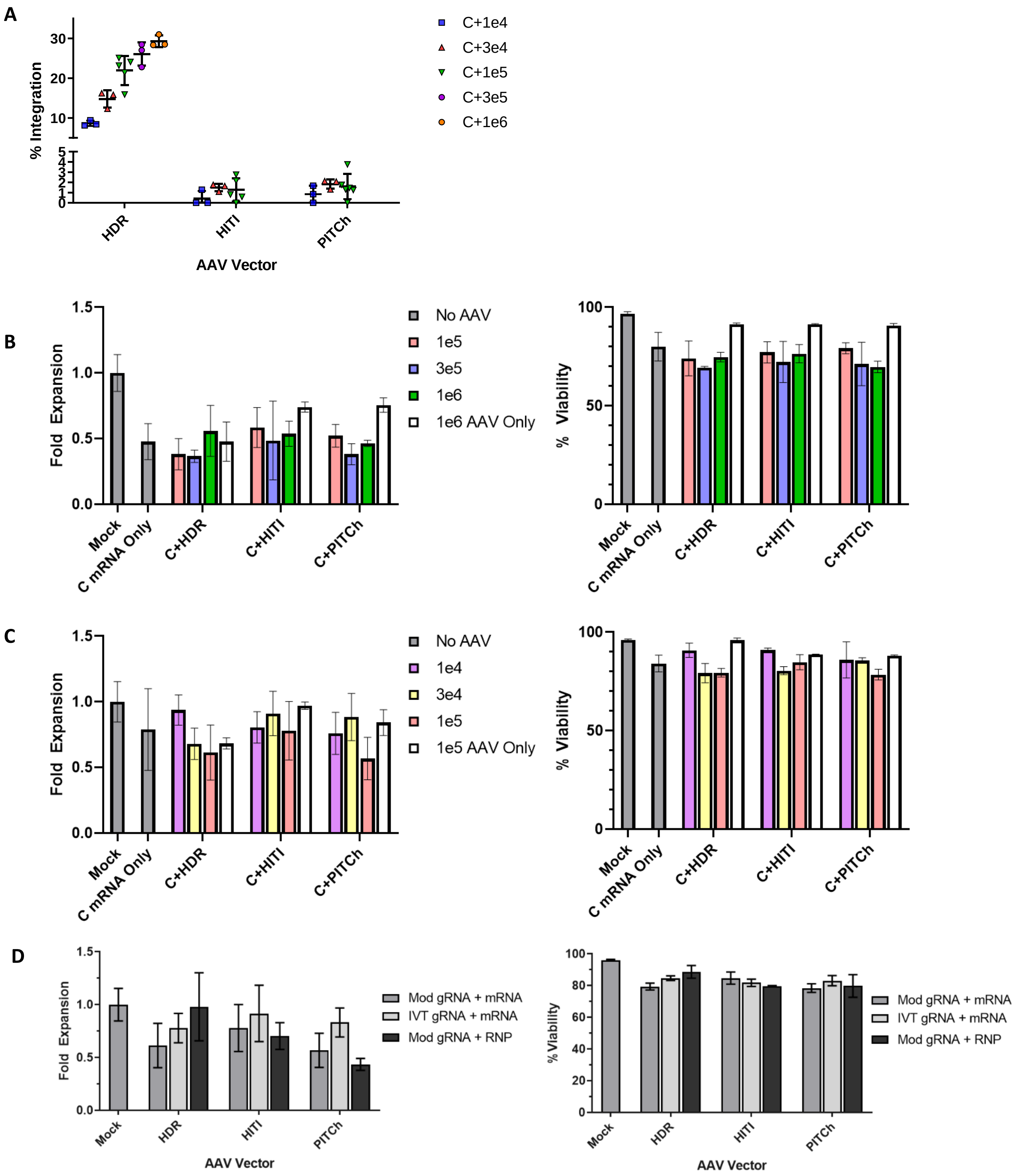

Supplemental Figure 5: Optimizing reagents for delivery into human CD34+ mobilized peripheral blood stem and progenitor cells

A) Human CD34+ cells taken from Granulocyte colony-stimulating factor mobilized peripheral blood were electroporated with Cas9 mRNA and single guide RNA (sgRNA) targeting BTK intron 1 before transduction with donor template adeno-associated viral vectors (AAVs) at various multiplicities of infections (MOIs). Data from two experiments combined (higher MOIs vs lower MOIs). **B)** Viability and fold expansion measured 1-day post electroporation. Donor templates AAVs were delivered at higher MOIs of 1e5, 3e5, or 1e6. **C)** Viability and fold expansion measured 1-day post electroporation. Donor templates AAVs were delivered at lower MOIs of 1e4, 3e4, or 1e5. **D)** Viability and fold expansion measured 1-day post electroporation from Fig 4A. Human PBSCs were treated with either *in vitro transcribed* (IVT) gRNA or chemically synthesized and modified (2'-O-methyl analogs and 3' phosphorothioate internucleotide linkages at each of the three 5' and 3' terminal RNA residues) gRNA. Cas9 was delivered either as mRNA or as RNP precomplexed with the respective gRNA. Cells were then transduced with donor AAV at an MOI of 1e5.

HDR Donor

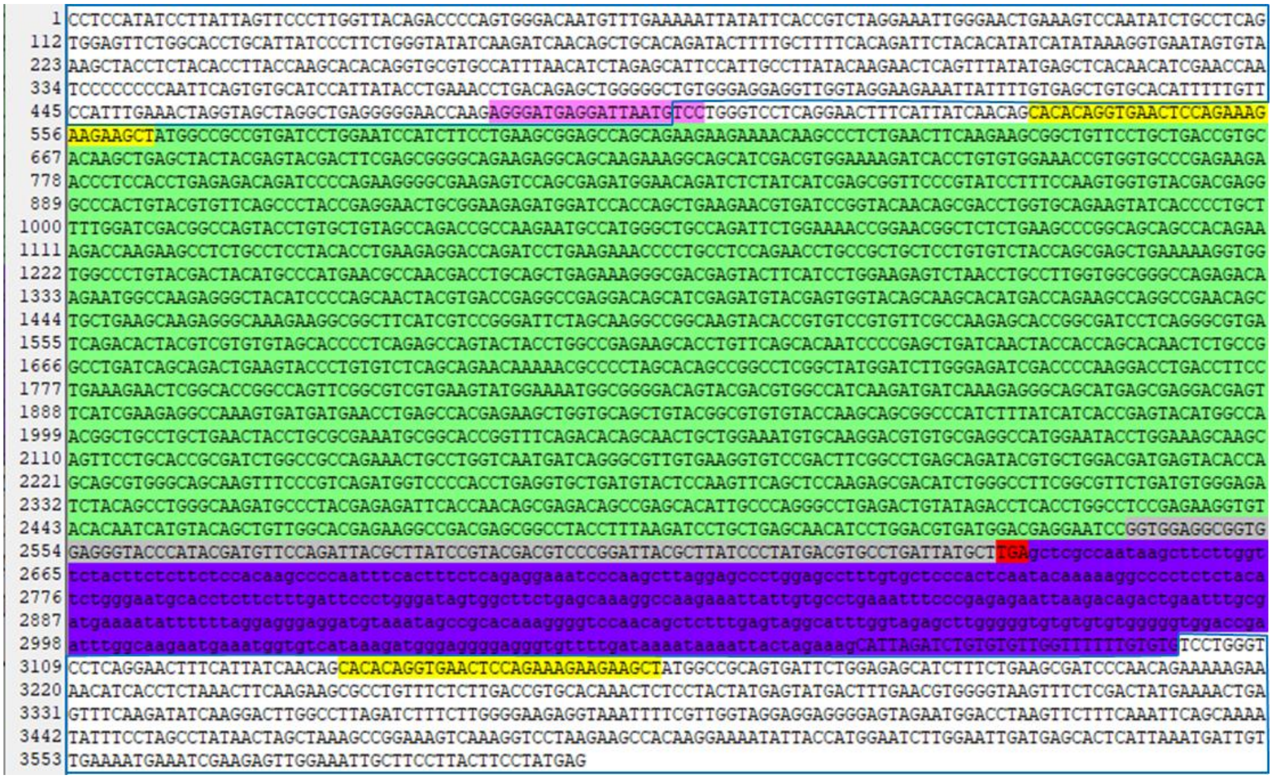

HDR ΔPAM Donor

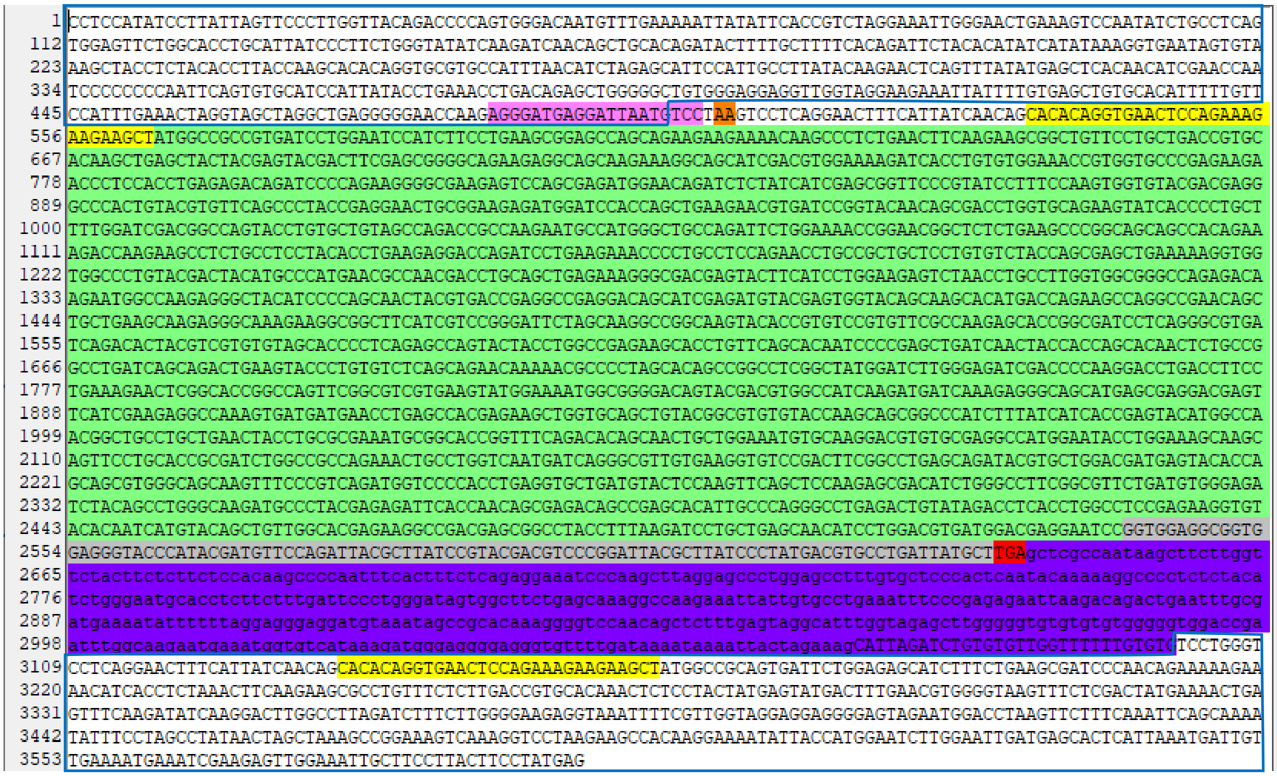

HITI-2c Donor

```
   1 CCAGGACATTAATCCTCATCCCTTCCTCAGGAACTTTCATTATCAACAGCACACAGGTGAACTCCAGAAAGAAGAAGCTATGGCCGCCGTGATCCTGGAATCCATCTTCCT
 112 GAAGCGGAGCCAGCAGAAGAAGAAAACAAGCCCTCTGAACTTCAAGAAGCGGCTGTTCCTGCTGACCGTGCACAAGCTGAGCTACTACGAGTACGACTTCGAGCGGGGCAG
 223 AAGAGGCAGCAAGAAAGGCAGCATCGACGTGGAAAAGATCACCTGTGTGGAAACCGTGGTGCCCGAGAAGAACCCTCCACCTGAGAGACAGATCCCCAGAAGGGGCGAAGA
 334 GTCCAGCGAGATGGAACAGATCTCTATCATCGAGCGGTTCCCGTATCCTTTCCAAGTGGTGTACGACGAGGGCCCACTGTACGTGTTCAGCCCTACCGAGGAACTGCGGAA
 445 GAGATGGATCCACCAGCTGAAGAACGTGATCCGGTACAACAGCGACCTGGTGCAGAAGTATCACCCCTGCTTTTGGATCGACGGCCAGTACCTGTGCTGTAGCCAGACCGC
 556 CAAGAATGCCATGGGCTGCCAGATTCTGGAAAACCGGAACGGCTCTCTGAAGCCCGGCAGCAGCCACAGAAAGACCAAGAAGCCTCTGCCTCCTACACCTGAAGAGGACCA
 667 GATCCTGAAGAAACCCCTGCCTCCAGAACCTGCCGCTGCTCCTGTGTCTACCAGCGAGCTGAAAAAGGTGGTGGCCCTGTACGACTACATGCCCATGAACGCCAACGACCT
 778 GCAGCTGAGAAAGGGCGACGAGTACTTCATCCTGGAAGAGTCTAACCTGCCTTGGTGGCGGGCCAGAGACAAGAATGGCCAAGAGGGCTACATCCCCAGCAACTACGTGAC
 889 CGAGGCCGAGGACAGCATCGAGATGTACGAGTGGTACAGCAAGCACATGACCAGAAGCCAGGCCGAACAGCTGCTGAAGCAAGAGGGCAAAGAAGGCGGCTTCATCGTCCG
1000 GGATTCTAGCAAGGCCGGCAAGTACACCGTGTCCGTGTTCGCCAAGAGCACCGGCGATCCTCAGGGCGTGATCAGACACTACGTCGTGTGTAGCACCCCTCAGAGCCAGTA
1111 CTACCTGGCCGAGAAGCACCTGTTCAGCACAATCCCCGAGCTGATCAACTACCACCAGCACAACTCTGCCGGCCTGATCAGCAGACTGAAGTACCCTGTGTCTCAGCAGAA
1222 CAAAAACGCCCCTAGCACAGCCGGCCTCGGCTATGGATCTTGGGAGATCGACCCCAAGGACCTGACCTTCCTGAAAGAACTCGGCACCGGCCAGTTCGGCGTCGTGAAGTA
1333 TGGAAAATGGCGGGGACAGTACGACGTGGCCATCAAGATGATCAAAGAGGGCAGCATGAGCGAGGACGAGTTCATCGAAGAGGCCAAAGTGATGATGAACCTGAGCCACGA
1444 GAAGCTGGTGCAGCTGTACGGCGTGTGTACCAAGCAGCGGCCCATCTTTATCATCACCGAGTACATGGCCAACGGCTGCCTGCTGAACTACCTGCGCGAAATGCGGCACCG
1555 GTTTCAGACACAGCAACTGCTGGAAATGTGCAAGGACGTGTGCGAGGCCATGGAATACCTGGAAAGCAAGCAGTTCCTGCACCGCGATCTGGCCGCCAGAAACTGCCTGGT
1666 CAATGATCAGGGCGTTGTGAAGGTGTCCGACTTCGGCCTGAGCAGATACGTGCTGGACGATGAGTACACCAGCAGCGTGGGCAGCAAGTTTCCCGTCAGATGGTCCCCACC
1777 TGAGGTGCTGATGTACTCCAAGTTCAGCTCCAAGAGCGACATCTGGGCCTTCGGCGTTCTGATGTGGGAGATCTACAGCCTGGGCAAGATGCCCTACGAGAGATTCACCAA
1888 CAGCGAGACAGCCGAGCACATTGCCCAGGGCCTGAGACTGTATAGACCTCACCTGGCCTCCGAGAAGGTGTACACAATCATGTACAGCTGTTGGCACGAGAAGGCCGACGA
1999 GCGGCCTACCTTTAAGATCCTGCTGAGCAACATCCTGGACGTGATGGACGAGGAATCCGGTGGAGGCGGTGGAGGGTACCCATACGATGTTCCAGATTACGCTTATCCGTA
2110 CGACGTCCCGGATTACGCTTATCCCTATGACGTGCCTGATTATGCTTGAgctcgccaataagcttcttggttctacttctcttctccacaagccccaatttcactttctca
2221 gaggaaatcccaagcttaggagccctggagcctttgtgctcccactcaatacaaaaaggcccctctctacatctgggaatgcacctcttctttgattccctgggatagtgg
2332 cttctgagcaaaggccaagaaattattgtgcctgaaatttcccgagagaattaagacagactgaatttgcgatgaaaatatttttaggagggaggatgtaaatagccgca
2443 caaaggggtccaacagctctttgagtaggcatttggtagagcttgggggtgtgtgtgtgggggtggaccgaatttggcaagaatgaaatggtgtcataaagatgggagggg
2554 agggtgttttgataaaataaaattactagaaagCATTAGATCTGTGTGTTGGTTTTTTGTGTGCCAGGACATTAATCCTCATCCCT
```

HITI-1c5’ Donor

```
   1 CCAGGACATTAATCCTCATCCCTTCCTCAGGAACTTTCATTATCAACAGCACACAGGTGAACTCCAGAAAGAAGAAGCTATGGCCGCCGTGATCCTGGAATCCATCTTCCTG
 113 AAGCGGAGCCAGCAGAAGAAGAAAACAAGCCCTCTGAACTTCAAGAAGCGGCTGTTCCTGCTGACCGTGCACAAGCTGAGCTACTACGAGTACGACTTCGAGCGGGGCAGAA
 225 GAGGCAGCAAGAAAGGCAGCATCGACGTGGAAAAGATCACCTGTGTGGAAACCGTGGTGCCCGAGAAGAACCCTCCACCTGAGAGACAGATCCCCAGAAGGGGCGAAGAGTC
 337 CAGCGAGATGGAACAGATCTCTATCATCGAGCGGTTCCCGTATCCTTTCCAAGTGGTGTACGACGAGGGCCCACTGTACGTGTTCAGCCCTACCGAGGAACTGCGGAAGAGA
 449 TGGATCCACCAGCTGAAGAACGTGATCCGGTACAACAGCGACCTGGTGCAGAAGTATCACCCCTGCTTTTGGATCGACGGCCAGTACCTGTGCTGTAGCCAGACCGCCAAGA
 561 ATGCCATGGGCTGCCAGATTCTGGAAAACCGGAACGGCTCTCTGAAGCCCGGCAGCAGCCACAGAAAGACCAAGAAGCCTCTGCCTCCTACACCTGAAGAGGACCAGATCCT
 673 GAAGAAACCCCTGCCTCCAGAACCTGCCGCTGCTCCTGTGTCTACCAGCGAGCTGAAAAAGGTGGTGGCCCTGTACGACTACATGCCCATGAACGCCAACGACCTGCAGCTG
 785 AGAAAGGGCGACGAGTACTTCATCCTGGAAGAGTCTAACCTGCCTTGGTGGCGGGCCAGAGACAAGAATGGCCAAGAGGGCTACATCCCCAGCAACTACGTGACCGAGGCCG
 897 AGGACAGCATCGAGATGTACGAGTGGTACAGCAAGCACATGACCAGAAGCCAGGCCGAACAGCTGCTGAAGCAAGAGGGCAAAGAAGGCGGCTTCATCGTCCGGGATTCTAG
1009 CAAGGCCGGCAAGTACACCGTGTCCGTGTTCGCCAAGAGCACCGGCGATCCTCAGGGCGTGATCAGACACTACGTCGTGTGTAGCACCCCTCAGAGCCAGTACTACCTGGCC
1121 GAGAAGCACCTGTTCAGCACAATCCCCGAGCTGATCAACTACCACCAGCACAACTCTGCCGGCCTGATCAGCAGACTGAAGTACCCTGTGTCTCAGCAGAACAAAAACGCCC
1233 CTAGCACAGCCGGCCTCGGCTATGGATCTTGGGAGATCGACCCCAAGGACCTGACCTTCCTGAAAGAACTCGGCACCGGCCAGTTCGGCGTCGTGAAGTATGGAAAATGGCG
1345 GGGACAGTACGACGTGGCCATCAAGATGATCAAAGAGGGCAGCATGAGCGAGGACGAGTTCATCGAAGAGGCCAAAGTGATGATGAACCTGAGCCACGAGAAGCTGGTGCAG
1457 CTGTACGGCGTGTGTACCAAGCAGCGGCCCATCTTTATCATCACCGAGTACATGGCCAACGGCTGCCTGCTGAACTACCTGCGCGAAATGCGGCACCGGTTTCAGACACAGC
1569 AACTGCTGGAAATGTGCAAGGACGTGTGCGAGGCCATGGAATACCTGGAAAGCAAGCAGTTCCTGCACCGCGATCTGGCCGCCAGAAACTGCCTGGTCAATGATCAGGGCGT
1681 TGTGAAGGTGTCCGACTTCGGCCTGAGCAGATACGTGCTGGACGATGAGTACACCAGCAGCGTGGGCAGCAAGTTTCCCGTCAGATGGTCCCCACCTGAGGTGCTGATGTAC
1793 TCCAAGTTCAGCTCCAAGAGCGACATCTGGGCCTTCGGCGTTCTGATGTGGGAGATCTACAGCCTGGGCAAGATGCCCTACGAGAGATTCACCAACAGCGAGACAGCCGAGC
1905 ACATTGCCCAGGGCCTGAGACTGTATAGACCTCACCTGGCCTCCGAGAAGGTGTACACAATCATGTACAGCTGTTGGCACGAGAAGGCCGACGAGCGGCCTACCTTTAAGAT
2017 CCTGCTGAGCAACATCCTGGACGTGATGGACGAGGAATCCGGTGGAGGCGGTGGAGGGTACCCATACGATGTTCCAGATTACGCTTATCCGTACGACGTCCCGGATTACGCT
2129 TATCCCTATGACGTGCCTGATTATGCTTGAgctcgccaataagcttcttggttctacttctcttctccacaagccccaatttcactttctcagaggaaatcccaagcttagg
2241 agccctggagcctttgtgctcccactcaatacaaaaaggcccctctctacatctgggaatgcacctcttctttgattccctgggatagtggcttctgagcaaaggccaagaa
2353 attattgtgcctgaaatttcccgagagaattaagacagactgaatttgcgatgaaaatatttttaggagggaggatgtaaatagccgcacaaaggggtccaacagctcttt
2465 gagtaggcatttggtagagcttgggggtgtgtgtgtgggggtggaccgaatttggcaagaatgaaatggtgtcataaagatgggaggggagggtgttttgataaaataaaat
2577 tactagaaagCATTAGATCTGTGTGTTGGTTTTTTGTGTG
```

HITI-1c3' Donor

```
   1 TCCTCAGGAACTTTCATTATCAACAGCACACAGGTGAACTCCAGAAAGAAGAAGCTATGGCCGCCGTGATCCTGGAATCCATCTTCCTGAAGCGGAGCCAGCAGAAGAAGAA
 113 AACAAGCCCTCTGAACTTCAAGAAGCGGCTGTTCCTGCTGACCGTGCACAAGCTGAGCTACTACGAGTACGACTTCGAGCGGGGCAGAAGAGGCAGCAAGAAAGGCAGCATC
 225 GACGTGGAAAAGATCACCTGTGTGGAAACCGTGGTGCCCGAGAAGAACCCTCCACCTGAGAGACAGATCCCCAGAAGGGGCGAAGAGTCCAGCGAGATGGAACAGATCTCTA
 337 TCATCGAGCGGTTCCCGTATCCTTTCCAAGTGGTGTACGACGAGGGCCCACTGTACGTGTTCAGCCCTACCGAGGAACTGCGGAAGAGATGGATCCACCAGCTGAAGAACGT
 449 GATCCGGTACAACAGCGACCTGGTGCAGAAGTATCACCCCTGCTTTTGGATCGACGGCCAGTACCTGTGCTGTAGCCAGACCGCCAAGAATGCCATGGGCTGCCAGATTCTG
 561 GAAAACCGGAACGGCTCTCTGAAGCCCGGCAGCAGCCACAGAAAGACCAAGAAGCCTCTGCCTCCTACACCTGAAGAGGACCAGATCCTGAAGAAACCCCTGCCTCCAGAAC
 673 CTGCCGCTGCTCCTGTGTCTACCAGCGAGCTGAAAAAGGTGGTGGCCCTGTACGACTACATGCCCATGAACGCCAACGACCTGCAGCTGAGAAAGGGCGACGAGTACTTCAT
 785 CCTGGAAGAGTCTAACCTGCCTTGGTGGCGGGCCAGAGACAAGAATGGCCAAGAGGGCTACATCCCCAGCAACTACGTGACCGAGGCCGAGGACAGCATCGAGATGTACGAG
 897 TGGTACAGCAAGCACATGACCAGAAGCCAGGCCGAACAGCTGCTGAAGCAAGAGGGCAAAGAAGGCGGCTTCATCGTCCGGGATTCTAGCAAGGCCGGCAAGTACACCGTGT
1009 CCGTGTTCGCCAAGAGCACCGGCGATCCTCAGGGCGTGATCAGACACTACGTCGTGTGTAGCACCCCTCAGAGCCAGTACTACCTGGCCGAGAAGCACCTGTTCAGCACAAT
1121 CCCCGAGCTGATCAACTACCACCAGCACAACTCTGCCGGCCTGATCAGCAGACTGAAGTACCCTGTGTCTCAGCAGAACAAAAACGCCCCTAGCACAGCCGGCCTCGGCTAT
1233 GGATCTTGGGAGATCGACCCCAAGGACCTGACCTTCCTGAAAGAACTCGGCACCGGCCAGTTCGGCGTCGTGAAGTATGGAAAATGGCGGGGACAGTACGACGTGGCCATCA
1345 AGATGATCAAAGAGGGCAGCATGAGCGAGGACGAGTTCATCGAAGAGGCCAAAGTGATGATGAACCTGAGCCACGAGAAGCTGGTGCAGCTGTACGGCGTGTGTACCAAGCA
1457 GCGGCCCATCTTTATCATCACCGAGTACATGGCCAACGGCTGCCTGCTGAACTACCTGCGCGAAATGCGGCACCGGTTTCAGACACAGCAACTGCTGGAAATGTGCAAGGAC
1569 GTGTGCGAGGCCATGGAATACCTGGAAAGCAAGCAGTTCCTGCACCGCGATCTGGCCGCCAGAAACTGCCTGGTCAATGATCAGGGCGTTGTGAAGGTGTCCGACTTCGGCC
1681 TGAGCAGATACGTGCTGGACGATGAGTACACCAGCAGCGTGGGCAGCAAGTTTCCCGTCAGATGGTCCCCACCTGAGGTGCTGATGTACTCCAAGTTCAGCTCCAAGAGCGA
1793 CATCTGGGCCTTCGGCGTTCTGATGTGGGAGATCTACAGCCTGGGCAAGATGCCCTACGAGAGATTCACCAACAGCGAGACAGCCGAGCACATTGCCCAGGGCCTGAGACTG
1905 TATAGACCTCACCTGGCCTCCGAGAAGGTGTACACAATCATGTACAGCTGTTGGCACGAGAAGGCCGACGAGCGGCCTACCTTTAAGATCCTGCTGAGCAACATCCTGGACG
2017 TGATGGACGAGGAATCCGGTGGAGGCGGTGGAGGGTACCCATACGATGTTCCAGATTACGCTTATCCGTACGACGTCCCGGATTACGCTTATCCCTATGACGTGCCTGATTA
2129 TGCTTGAgctcgccaataagcttcttggttctacttctcttctccacaagccccaatttcactttctcagaggaaatcccaagcttaggagccctggagcctttgtgctccc
2241 actcaatacaaaaaggcccctctctacatctgggaatgcacctcttctttgattccctgggatagtggcttctgagcaaaggccaagaaattattgtgcctgaaatttcccg
2353 agagaattaagacagactgaatttgcgatgaaaatatttttttaggagggaggatgtaaatagccgcacaaaggggtccaacagctctttgagtaggcatttggtagagcttg
2465 ggggtgtgtgtgtgggggtggaccgaatttggcaagaatgaaatggtgtcataaagatgggaggggagggtgttttgataaaataaaattactagaaagCATTAGATCTGTG
2577 TGTTGGTTTTTGTGTGCCAGGACATTAATCCTCATCCCT
```

HITI-0c Donor

```
   1 TCCTCAGGAACTTTCATTATCAACAGCACACAGGTGAACTCCAGAAAGAAGAAGCTATGGCCGCCGTGATCCTGGAATCCATCTTCCTGAAGCGGAGCCAGCAGAAGAAGAA
 113 AACAAGCCCTCTGAACTTCAAGAAGCGGCTGTTCCTGCTGACCGTGCACAAGCTGAGCTACTACGAGTACGACTTCGAGCGGGGCAGAAGAGGCAGCAAGAAAGGCAGCATC
 225 GACGTGGAAAAGATCACCTGTGTGGAAACCGTGGTGCCCGAGAAGAACCCTCCACCTGAGAGACAGATCCCCAGAAGGGGCGAAGAGTCCAGCGAGATGGAACAGATCTCTA
 337 TCATCGAGCGGTTCCCGTATCCTTTCCAAGTGGTGTACGACGAGGGCCCACTGTACGTGTTCAGCCCTACCGAGGAACTGCGGAAGAGATGGATCCACCAGCTGAAGAACGT
 449 GATCCGGTACAACAGCGACCTGGTGCAGAAGTATCACCCCTGCTTTTGGATCGACGGCCAGTACCTGTGCTGTAGCCAGACCGCCAAGAATGCCATGGGCTGCCAGATTCTG
 561 GAAAACCGGAACGGCTCTCTGAAGCCCGGCAGCAGCCACAGAAAGACCAAGAAGCCTCTGCCTCCTACACCTGAAGAGGACCAGATCCTGAAGAAACCCCTGCCTCCAGAAC
 673 CTGCCGCTGCTCCTGTGTCTACCAGCGAGCTGAAAAAGGTGGTGGCCCTGTACGACTACATGCCCATGAACGCCAACGACCTGCAGCTGAGAAAGGGCGACGAGTACTTCAT
 785 CCTGGAAGAGTCTAACCTGCCTTGGTGGCGGGCCAGAGACAAGAATGGCCAAGAGGGCTACATCCCCAGCAACTACGTGACCGAGGCCGAGGACAGCATCGAGATGTACGAG
 897 TGGTACAGCAAGCACATGACCAGAAGCCAGGCCGAACAGCTGCTGAAGCAAGAGGGCAAAGAAGGCGGCTTCATCGTCCGGGATTCTAGCAAGGCCGGCAAGTACACCGTGT
1009 CCGTGTTCGCCAAGAGCACCGGCGATCCTCAGGGCGTGATCAGACACTACGTCGTGTGTAGCACCCCTCAGAGCCAGTACTACCTGGCCGAGAAGCACCTGTTCAGCACAAT
1121 CCCCGAGCTGATCAACTACCACCAGCACAACTCTGCCGGCCTGATCAGCAGACTGAAGTACCCTGTGTCTCAGCAGAACAAAAACGCCCCTAGCACAGCCGGCCTCGGCTAT
1233 GGATCTTGGGAGATCGACCCCAAGGACCTGACCTTCCTGAAAGAACTCGGCACCGGCCAGTTCGGCGTCGTGAAGTATGGAAAATGGCGGGGACAGTACGACGTGGCCATCA
1345 AGATGATCAAAGAGGGCAGCATGAGCGAGGACGAGTTCATCGAAGAGGCCAAAGTGATGATGAACCTGAGCCACGAGAAGCTGGTGCAGCTGTACGGCGTGTGTACCAAGCA
1457 GCGGCCCATCTTTATCATCACCGAGTACATGGCCAACGGCTGCCTGCTGAACTACCTGCGCGAAATGCGGCACCGGTTTCAGACACAGCAACTGCTGGAAATGTGCAAGGAC
1569 GTGTGCGAGGCCATGGAATACCTGGAAAGCAAGCAGTTCCTGCACCGCGATCTGGCCGCCAGAAACTGCCTGGTCAATGATCAGGGCGTTGTGAAGGTGTCCGACTTCGGCC
1681 TGAGCAGATACGTGCTGGACGATGAGTACACCAGCAGCGTGGGCAGCAAGTTTCCCGTCAGATGGTCCCCACCTGAGGTGCTGATGTACTCCAAGTTCAGCTCCAAGAGCGA
1793 CATCTGGGCCTTCGGCGTTCTGATGTGGGAGATCTACAGCCTGGGCAAGATGCCCTACGAGAGATTCACCAACAGCGAGACAGCCGAGCACATTGCCCAGGGCCTGAGACTG
1905 TATAGACCTCACCTGGCCTCCGAGAAGGTGTACACAATCATGTACAGCTGTTGGCACGAGAAGGCCGACGAGCGGCCTACCTTTAAGATCCTGCTGAGCAACATCCTGGACG
2017 TGATGGACGAGGAATCCGGTGGAGGCGGTGGAGGGTACCCATACGATGTTCCAGATTACGCTTATCCGTACGACGTCCCGGATTACGCTTATCCCTATGACGTGCCTGATTA
2129 TGCTTGAgctcgccaataagcttcttggttctacttctcttctccacaagccccaatttcactttctcagaggaaatcccaagcttaggagccctggagcctttgtgctccc
2241 actcaatacaaaaaggcccctctctacatctgggaatgcacctcttctttgattccctgggatagtggcttctgagcaaaggccaagaaattattgtgcctgaaatttcccg
2353 agagaattaagacagactgaatttgcgatgaaaatatttttttaggagggaggatgtaaatagccgcacaaaggggtccaacagctctttgagtaggcatttggtagagcttg
2465 ggggtgtgtgtgtgggggtggaccgaatttggcaagaatgaaatggtgtcataaagatgggaggggagggtgttttgataaaataaaattactagaaagCATTAGATCTGTG
2577 TGTTGGTTTTTGTGTG
```

PITCh-1 Donor

```
   1 AGGGATGAGGATTAATGTCCTGGAAGAGGGATGAGGATTAATGTCCTCAGGAACTTTCATTATCAACAGCACACAGGTGAACTCCAGAAAGAAGAAGCTATGGCCGCCGTG
 112 ATCCTGGAATCCATCTTCCTGAAGCGGAGCCAGCAGAAGAAGAAAACAAGCCCTCTGAACTTCAAGAAGCGGCTGTTCCTGCTGACCGTGCACAAGCTGAGCTACTACGAG
 223 TACGACTTCGAGCGGGGCAGAAGAGGCAGCAAGAAAGGCAGCATCGACGTGGAAAAGATCACCTGTGTGGAAACCGTGGTGCCCGAGAAGAACCCTCCACCTGAGAGACAG
 334 ATCCCCAGAAGGGGCGAAGAGTCCAGCGAGATGGAACAGATCTCTATCATCGAGCGGTTCCCGTATCCTTTCCAAGTGGTGTACGACGAGGGCCCACTGTACGTGTTCAGC
 445 CCTACCGAGGAACTGCGGAAGAGATGGATCCACCAGCTGAAGAACGTGATCCGGTACAACAGCGACCTGGTGCAGAAGTATCACCCCTGCTTTTGGATCGACGGCCAGTAC
 556 CTGTGCTGTAGCCAGACCGCCAAGAATGCCATGGGCTGCCAGATTCTGGAAAACCGGAACGGCTCTCTGAAGCCCGGCAGCAGCCACAGAAAGACCAAGAAGCCTCTGCCT
 667 CCTACACCTGAAGAGGACCAGATCCTGAAGAAACCCCTGCCTCCAGAACCTGCCGCTGCTCCTGTGTCTACCAGCGAGCTGAAAAAGGTGGTGGCCCTGTACGACTACATG
 778 CCCATGAACGCCAACGACCTGCAGCTGAGAAAGGGCGACGAGTACTTCATCCTGGAAGAGTCTAACCTGCCTTGGTGGCGGGCCAGAGACAAGAATGGCCAAGAGGGCTAC
 889 ATCCCCAGCAACTACGTGACCGAGGCCGAGGACAGCATCGAGATGTACGAGTGGTACAGCAAGCACATGACCAGAAGCCAGGCCGAACAGCTGCTGAAGCAAGAGGGCAAA
1000 GAAGGCGGCTTCATCGTCCGGGATTCTAGCAAGGCCGGCAAGTACACCGTGTCCGTGTTCGCCAAGAGCACCGGCGATCCTCAGGGCGTGATCAGACACTACGTCGTGTGT
1111 AGCACCCCTCAGAGCCAGTACTACCTGGCCGAGAAGCACCTGTTCAGCACAATCCCCGAGCTGATCAACTACCACCAGCACAACTCTGCCGGCCTGATCAGCAGACTGAAG
1222 TACCCTGTGTCTCAGCAGAACAAAAACGCCCCTAGCACAGCCGGCCTCGGCTATGGATCTTGGGAGATCGACCCCAAGGACCTGACCTTCCTGAAAGAACTCGGCACCGGC
1333 CAGTTCGGCGTCGTGAAGTATGGAAAATGGCGGGGACAGTACGACGTGGCCATCAAGATGATCAAAGAGGGCAGCATGAGCGAGGACGAGTTCATCGAAGAGGCCAAAGTG
1444 ATGATGAACCTGAGCCACGAGAAGCTGGTGCAGCTGTACGGCGTGTGTACCAAGCAGCGGCCCATCTTTATCATCACCGAGTACATGGCCAACGGCTGCCTGCTGAACTAC
1555 CTGCGCGAAATGCGGCACCGGTTTCAGACACAGCAACTGCTGGAAATGTGCAAGGACGTGTGCGAGGCCATGGAATACCTGGAAAGCAAGCAGTTCCTGCACCGCGATCTG
1666 GCCGCCAGAAACTGCCTGGTCAATGATCAGGGCGTTGTGAAGGTGTCCGACTTCGGCCTGAGCAGATACGTGCTGGACGATGAGTACACCAGCAGCGTGGGCAGCAAGTTT
1777 CCCGTCAGATGGTCCCCACCTGAGGTGCTGATGTACTCCAAGTTCAGCTCCAAGAGCGACATCTGGGCCTTCGGCGTTCTGATGTGGGAGATCTACAGCCTGGGCAAGATG
1888 CCCTACGAGAGATTCACCAACAGCGAGACAGCCGAGCACATTGCCCAGGGCCTGAGACTGTATAGACCTCACCTGGCCTCCGAGAAGGTGTACACAATCATGTACAGCTGT
1999 TGGCACGAGAAGGCCGACGAGCGGCCTACCTTTAAGATCCTGCTGAGCAACATCCTGGACGTGATGGACGAGGAATCCGGTGGAGGCGGTGGAGGGTACCCATACGATGTT
2110 CCAGATTACGCTTATCCGTACGACGTCCCGGATTACGCTTATCCCTATGACGTGCCTGATTATGCTTGAgctcgccaataagcttcttggttctacttctcttctccacaa
2221 gccccaatttcactttctcagaggaaatcccaagcttaggagccctggagcctttgtgctcccactcaatacaaaaaggcccctctctacatctgggaatgcacctcttct
2332 ttgattccctgggatagtggcttctgagcaaaggccaagaaattattgtgcctgaaatttcccgagagaattaagacagactgaatttgcgatgaaaatattttttaggag
2443 ggaggatgtaaatagccgcacaaaggggtccaacagctctttgagtaggcatttggtagagcttgggggtgtgtgtgtgggggtggaccgaatttggcaagaatgaaatgg
2554 tgtcataaagatgggaggggagggtgttttgataaaataaaattactagaaagCATTAGATCTGTGTGTTGGTTTTTTGTGTGTCCTGGGTCCTCAGGAACTTCCAGGACA
2665 TTAATCCTCATCCCT
```

PITCh-2 Donor

```
   1 AGGGATGAGGATTAATGTCCTGGAAGAGGGATGAGGATTAATGTCCTCAGGAACTTTCATTATCAACAGCACACAGGTGAACTCCAGAAAGAAGAAGCTATGGCCGCCGTG
 112 ATCCTGGAATCCATCTTCCTGAAGCGGAGCCAGCAGAAGAAGAAAACAAGCCCTCTGAACTTCAAGAAGCGGCTGTTCCTGCTGACCGTGCACAAGCTGAGCTACTACGAG
 223 TACGACTTCGAGCGGGGCAGAAGAGGCAGCAAGAAAGGCAGCATCGACGTGGAAAAGATCACCTGTGTGGAAACCGTGGTGCCCGAGAAGAACCCTCCACCTGAGAGACAG
 334 ATCCCCAGAAGGGGCGAAGAGTCCAGCGAGATGGAACAGATCTCTATCATCGAGCGGTTCCCGTATCCTTTCCAAGTGGTGTACGACGAGGGCCCACTGTACGTGTTCAGC
 445 CCTACCGAGGAACTGCGGAAGAGATGGATCCACCAGCTGAAGAACGTGATCCGGTACAACAGCGACCTGGTGCAGAAGTATCACCCCTGCTTTTGGATCGACGGCCAGTAC
 556 CTGTGCTGTAGCCAGACCGCCAAGAATGCCATGGGCTGCCAGATTCTGGAAAACCGGAACGGCTCTCTGAAGCCCGGCAGCAGCCACAGAAAGACCAAGAAGCCTCTGCCT
 667 CCTACACCTGAAGAGGACCAGATCCTGAAGAAACCCCTGCCTCCAGAACCTGCCGCTGCTCCTGTGTCTACCAGCGAGCTGAAAAAGGTGGTGGCCCTGTACGACTACATG
 778 CCCATGAACGCCAACGACCTGCAGCTGAGAAAGGGCGACGAGTACTTCATCCTGGAAGAGTCTAACCTGCCTTGGTGGCGGGCCAGAGACAAGAATGGCCAAGAGGGCTAC
 889 ATCCCCAGCAACTACGTGACCGAGGCCGAGGACAGCATCGAGATGTACGAGTGGTACAGCAAGCACATGACCAGAAGCCAGGCCGAACAGCTGCTGAAGCAAGAGGGCAAA
1000 GAAGGCGGCTTCATCGTCCGGGATTCTAGCAAGGCCGGCAAGTACACCGTGTCCGTGTTCGCCAAGAGCACCGGCGATCCTCAGGGCGTGATCAGACACTACGTCGTGTGT
1111 AGCACCCCTCAGAGCCAGTACTACCTGGCCGAGAAGCACCTGTTCAGCACAATCCCCGAGCTGATCAACTACCACCAGCACAACTCTGCCGGCCTGATCAGCAGACTGAAG
1222 TACCCTGTGTCTCAGCAGAACAAAAACGCCCCTAGCACAGCCGGCCTCGGCTATGGATCTTGGGAGATCGACCCCAAGGACCTGACCTTCCTGAAAGAACTCGGCACCGGC
1333 CAGTTCGGCGTCGTGAAGTATGGAAAATGGCGGGGACAGTACGACGTGGCCATCAAGATGATCAAAGAGGGCAGCATGAGCGAGGACGAGTTCATCGAAGAGGCCAAAGTG
1444 ATGATGAACCTGAGCCACGAGAAGCTGGTGCAGCTGTACGGCGTGTGTACCAAGCAGCGGCCCATCTTTATCATCACCGAGTACATGGCCAACGGCTGCCTGCTGAACTAC
1555 CTGCGCGAAATGCGGCACCGGTTTCAGACACAGCAACTGCTGGAAATGTGCAAGGACGTGTGCGAGGCCATGGAATACCTGGAAAGCAAGCAGTTCCTGCACCGCGATCTG
1666 GCCGCCAGAAACTGCCTGGTCAATGATCAGGGCGTTGTGAAGGTGTCCGACTTCGGCCTGAGCAGATACGTGCTGGACGATGAGTACACCAGCAGCGTGGGCAGCAAGTTT
1777 CCCGTCAGATGGTCCCCACCTGAGGTGCTGATGTACTCCAAGTTCAGCTCCAAGAGCGACATCTGGGCCTTCGGCGTTCTGATGTGGGAGATCTACAGCCTGGGCAAGATG
1888 CCCTACGAGAGATTCACCAACAGCGAGACAGCCGAGCACATTGCCCAGGGCCTGAGACTGTATAGACCTCACCTGGCCTCCGAGAAGGTGTACACAATCATGTACAGCTGT
1999 TGGCACGAGAAGGCCGACGAGCGGCCTACCTTTAAGATCCTGCTGAGCAACATCCTGGACGTGATGGACGAGGAATCCGGTGGAGGCGGTGGAGGGTACCCATACGATGTT
2110 CCAGATTACGCTTATCCGTACGACGTCCCGGATTACGCTTATCCCTATGACGTGCCTGATTATGCTTGAgctcgccaataagcttcttggttctacttctcttctccacaa
2221 gccccaatttcactttctcagaggaaatcccaagcttaggagccctggagcctttgtgctcccactcaatacaaaaaggcccctctctacatctgggaatgcacctcttct
2332 ttgattccctgggatagtggcttctgagcaaaggccaagaaattattgtgcctgaaatttcccgagagaattaagacagactgaatttgcgatgaaaatattttttaggag
2443 ggaggatgtaaatagccgcacaaaggggtccaacagctctttgagtaggcatttggtagagcttgggggtgtgtgtgtgggggtggaccgaatttggcaagaatgaaatgg
2554 tgtcataaagatgggaggggagggtgttttgataaaataaaattactagaaagCATTAGATCTGTGTGTTGGTTTTTTGTGTGTCCTGGGTCCTCAGGAACTTAGGGATGA
2665 GGATTAATGTCCTGG
```

PITCH-0c Donor

```
   1 AAGAGGGATGAGGATTAATGTCCTCAGGAACTTTCATTATCAACAGCACACAGGTGAACTCCAGAAAGAAGAAGCTATGGCCGCCGTGATCCTGGAATCCATCTTCCTGAA
 112 GCGGAGCCAGCAGAAGAAGAAAACAAGCCCTCTGAACTTCAAGAAGCGGCTGTTCCTGCTGACCGTGCACAAGCTGAGCTACTACGAGTACGACTTCGAGCGGGGCAGAAG
 223 AGGCAGCAAGAAAGGCAGCATCGACGTGGAAAAGATCACCTGTGTGGAAACCGTGGTGCCCGAGAAGAACCCTCCACCTGAGAGACAGATCCCCAGAAGGGGCGAAGAGTC
 334 CAGCGAGATGGAACAGATCTCTATCATCGAGCGGTTCCCGTATCCTTTCCAAGTGGTGTACGACGAGGGCCCACTGTACGTGTTCAGCCCTACCGAGGAACTGCGGAAGAG
 445 ATGGATCCACCAGCTGAAGAACGTGATCCGGTACAACAGCGACCTGGTGCAGAAGTATCACCCCTGCTTTTGGATCGACGGCCAGTACCTGTGCTGTAGCCAGACCGCCAA
 556 GAATGCCATGGGCTGCCAGATTCTGGAAAACCGGAACGGCTCTCTGAAGCCCGGCAGCAGCCACAGAAAGACCAAGAAGCCTCTGCCTCCTACACCTGAAGAGGACCAGAT
 667 CCTGAAGAAACCCCTGCCTCCAGAACCTGCCGCTGCTCCTGTGTCTACCAGCGAGCTGAAAAAGGTGGTGGCCCTGTACGACTACATGCCCATGAACGCCAACGACCTGCA
 778 GCTGAGAAAGGGCGACGAGTACTTCATCCTGGAAGAGTCTAACCTGCCTTGGTGGCGGGCCAGAGACAAGAATGGCCAAGAGGGCTACATCCCCAGCAACTACGTGACCGA
 889 GGCCGAGGACAGCATCGAGATGTACGAGTGGTACAGCAAGCACATGACCAGAAGCCAGGCCGAACAGCTGCTGAAGCAAGAGGGCAAAGAAGGCGGCTTCATCGTCCGGGA
1000 TTCTAGCAAGGCCGGCAAGTACACCGTGTCCGTGTTCGCCAAGAGCACCGGCGATCCTCAGGGCGTGATCAGACACTACGTCGTGTGTAGCACCCCTCAGAGCCAGTACTA
1111 CCTGGCCGAGAAGCACCTGTTCAGCACAATCCCCGAGCTGATCAACTACCACCAGCACAACTCTGCCGGCCTGATCAGCAGACTGAAGTACCCTGTGTCTCAGCAGAACAA
1222 AAACGCCCCTAGCACAGCCGGCCTCGGCTATGGATCTTGGGAGATCGACCCCAAGGACCTGACCTTCCTGAAAGAACTCGGCACCGGCCAGTTCGGCGTCGTGAAGTATGG
1333 AAAATGGCGGGGACAGTACGACGTGGCCATCAAGATGATCAAAGAGGGCAGCATGAGCGAGGACGAGTTCATCGAAGAGGCCAAAGTGATGATGAACCTGAGCCACGAGAA
1444 GCTGGTGCAGCTGTACGGCGTGTGTACCAAGCAGCGGCCCATCTTTATCATCACCGAGTACATGGCCAACGGCTGCCTGCTGAACTACCTGCGCGAAATGCGGCACCGGTT
1555 TCAGACACAGCAACTGCTGGAAATGTGCAAGGACGTGTGCGAGGCCATGGAATACCTGGAAAGCAAGCAGTTCCTGCACCGCGATCTGGCCGCCAGAAACTGCCTGGTCAA
1666 TGATCAGGGCGTTGTGAAGGTGTCCGACTTCGGCCTGAGCAGATACGTGCTGGACGATGAGTACACCAGCAGCGTGGGCAGCAAGTTTCCCGTCAGATGGTCCCCACCTGA
1777 GGTGCTGATGTACTCCAAGTTCAGCTCCAAGAGCGACATCTGGGCCTTCGGCGTTCTGATGTGGGAGATCTACAGCCTGGGCAAGATGCCCTACGAGAGATTCACCAACAG
1888 CGAGACAGCCGAGCACATTGCCCAGGGCCTGAGACTGTATAGACCTCACCTGGCCTCCGAGAAGGTGTACACAATCATGTACAGCTGTTGGCACGAGAAGGCCGACGAGCG
1999 GCCTACCTTTAAGATCCTGCTGAGCAACATCCTGGACGTGATGGACGAGGAATCCGGTGGAGGCGGTGGAGGGTACCCATACGATGTTCCAGATTACGCTTATCCGTACGA
2110 CGTCCCGGATTACGCTTATCCCTATGACGTGCCTGATTATGCTTGAgctcgccaataagcttcttggttctacttctcttctccacaagccccaatttcactttctcagag
2221 gaaatcccaagcttaggagccctggagcctttgtgctcccactcaatacaaaaaggcccctctctacatctgggaatgcacctcttctttgattccctgggatagtggctt
2332 ctgagcaaaggccaagaaattattgtgcctgaaatttcccgagagaattaagacagactgaatttgcgatgaaaatatttttttaggagggaggatgtaaatagccgcacaa
2443 aggggtccaacagctctttgagtaggcatttggtagagcttgggggtgtgtgtgtgggggtggaccgaatttggcaagaatgaaatggtgtcataaagatgggaggggagg
2554 gtgttttgataaaataaaattactagaaagCATTAGATCTGTGTGTTGGTTTTTTGTGTGTCCTGGGTCCTCAGGAACTT
```

Supplemental Figure 6: Annotated schematics of each of the nine donors that were cloned into the TOPO-2.1 plasmid backbone or an Adeno Associated Viral vector packaging plasmid backbone. The protospacer sequence is represented by pink. Homology arms are enclosed in a blue box. Yellow highlighting represents the non-coding portion of BTK exon 2. The coding regions of the BTK cDNA (exons 2-19) are in green. The 3 C terminal hemagglutinin tags and linker sequence are in gray, immediately preceding the stop codon in red. The 3’ UTR sequence is in purple.
